# Facebook Intervention for Young-Onset Melanoma Survivors and Families: Protocol for a Randomized Controlled Trial

**DOI:** 10.2196/39640

**Published:** 2023-01-24

**Authors:** Sharon Manne, Sherry Pagoto, Susan Peterson, Carolyn Heckman, Deborah Kashy, Adam Berger, Christina Studts, Rosalyn Negrón, David Buller, Lisa Paddock, Joseph Gallo, Alexandria Kulik, Sara Frederick, Morgan Pesanelli, Mara Domider, Marissa Grosso

**Affiliations:** 1 Behavioral Sciences Rutgers Cancer Institute of New Jersey Rutgers, The State University of New Jersey New Brunswick, NJ United States; 2 Department of Allied Health Sciences College of Agriculture, Health and Natural Resources University of Connecticut Storrs, CT United States; 3 Department of Behavioral Science, Division of Cancer Prevention and Population Sciences MD Anderson Cancer Center The University of Texas San Antonio, TX United States; 4 College of Social Science Department of Psychology Michigan State University East Lansing, MI United States; 5 Division of Surgical Oncology, Department of Surgery Robert Wood Johnson Medical School Rutgers, The State University of New Jersey New Brunswick, NJ United States; 6 Pediatrics - General Pediatrics School of Medicine University of Colorado Anschutz Medical Campus Aurora, CO United States; 7 College of Liberal Arts University of Massachusetts Boston Boston, MA United States; 8 Klein Buendel, Inc Golden, CO United States; 9 Rutgers Cancer Institute of New Jersey New Brunswick, NJ United States; 10 Hackensack Meridian Jersey Shore Medical Center Hackensack Meridian Health Neptune City, NJ United States; 11 School of Public Health Rutgers, The State University of New Jersey New Brunswick, NJ United States

**Keywords:** cancer survivors, melanoma survivors, skin self-examination, clinical skin examination, sun protection, behavioral intervention, social media

## Abstract

**Background:**

Individuals diagnosed with melanoma before the age of 40 years (young-onset melanoma survivors) and their first-degree relatives (FDRs) are a growing population at risk for developing recurrent melanoma or new melanomas. Regular surveillance using clinical skin examination (CSE) and skin self-examination (SSE) and engagement in preventive behaviors including sun protection are recommended. Given the growing population of survivors and their families who are at increased risk, it is surprising that no behavioral interventions have been developed and evaluated to improve risk-reduction behaviors.

**Objective:**

We describe the rationale and methodology for a randomized controlled trial evaluating the efficacy of a Facebook intervention providing information, goal setting, and peer support to increase CSE, SSE, and sun protection for young-onset melanoma survivors and their FDRs.

**Methods:**

Overall, 577 survivors and 577 FDRs will be randomly assigned to either the Young Melanoma Family Facebook Group or the Melanoma Family Healthy Lifestyle Facebook Group condition. Participants will complete measures of CSE, SSE, and sun protection, and mediator measures of attitudes and beliefs before and after the intervention. The primary aim is to evaluate the impact of the Young Melanoma Family Facebook intervention versus the Melanoma Family Healthy Lifestyle Facebook intervention on CSE, SSE frequency and comprehensiveness, and sun protection among FDRs. The secondary aims examine the efficacy of the Young Melanoma Family Facebook intervention on survivors’ SSE frequency and comprehensiveness and sun protection behaviors and mechanisms of intervention efficacy for intervention impact on FDR and survivor outcomes. The exploratory aim is to evaluate the efficacy of the 2 interventions on perceived stress, physical activity, and healthy eating.

**Results:**

This project was funded by the National Institutes of Health (R01CA221854). The project began in May 2018, and recruitment started in January 2019. We anticipate completing enrollment by November 2023. Power calculations recommended a sample size of 577 survivors and 577 FDRs. Multilevel modeling treating family as the upper-level sampling unit and individual as the lower-level sampling unit will be the primary data analytic approach. Fixed effect predictors in these models will include condition, role, sex, all 2- and 3-way interactions, and covariates.

**Conclusions:**

The Young Melanoma Family Facebook intervention aims to improve primary and secondary skin cancer prevention for young-onset melanoma survivors and their family members. The intervention’s delivery via a popular, freely available social media platform increases its impact because of the potential for dissemination in many contexts. If efficacious, this program could be disseminated by dermatologist practices, public health or nonprofit organizations focused on melanoma, and existing melanoma and skin cancer Facebook groups, thereby expanding its reach. This project will produce a content library of posts and a moderation guide for others.

**Trial Registration:**

ClinicalTrials.gov NCT03677739; https://clinicaltrials.gov/ct2/show/NCT03677739

**International Registered Report Identifier (IRRID):**

DERR1-10.2196/39640

## Introduction

### Background

Young adults diagnosed with melanoma, defined as individuals diagnosed at or before the age of 39 years, have been identified by the National Cancer Institute as a growing population [1]. Melanoma risk is ≥6 times higher among young adults than it was 40 years ago [2] and is the most common malignancy for young adults aged between 25 and 29 years [3]. Young adult survivors are at a 9-fold risk for developing another melanoma and a higher relative risk for a second malignancy than adults diagnosed with cancer who are >39 years of age [4]. First-degree relatives (FDRs) of patients diagnosed with melanoma before the age of 40 years are also at an elevated risk of developing melanoma. Having an FDR with melanoma more than doubles the risk of melanoma [5].

The incidence of a second melanoma is increased in melanoma survivors, with cumulative risk ranging from 2% to 5% during the periods of 5 to 20 years after initial diagnosis [6-11]. Existing guidelines vary only slightly among specialty organizations. The National Comprehensive Cancer Network recommends lifelong clinical skin examination (CSE) at least annually for those with early-stage disease and more frequent follow-ups for the first 2 years for those with later stage disease (ie, every 3-6 months) [12]. The National Comprehensive Cancer Network guidelines and other professional agencies also recommend education in regular skin self-examination (SSE) and principles of sun safety exposure (avoidance of sun during peak hours or use of sun protective clothing, hat, or eyewear) [12-14]. Similarly, many professional societies suggest routine CSE and SSE and regular sun protection behaviors for FDRs [11,15].

Despite the growing population of young melanoma survivors and their FDRs, engagement in CSE, SSE, and sun protection behaviors in this population remains low. Bergenmar and Brandberg [16] evaluated young adults with a family history of melanoma and found that engagement in sun protection was low and levels of sun exposure were high. Zwemer et al [14] evaluated sun protection practices among melanoma survivors <30 years and reported low levels of sun protection in one-third of the sample. More than half of the participants reported sunbathing for >1 hour a week. Our preliminary data suggest that engagement in sun protection, SSE, and CSE is also relatively low among FDRs. Approximately 44% of FDRs never had a CSE, 73% had not conducted a comprehensive SSE in the past 2 months, and 33% had rarely or never used sunscreen or a wide-brimmed hat when outdoors during daytime hours (SL Manne, PhD, unpublished data, March 2019).

Given the growing population of young melanoma survivors and their family members who are at increased risk, it is surprising that no behavioral interventions have been developed and evaluated to improve risk-reduction behaviors in this population. Behavioral interventions have been evaluated to improve sun protection and skin surveillance among melanoma survivors and FDRs of patients of all ages [17-19], as well as SSEs among patients with melanoma of all ages [20-23]. However, no study has specifically targeted the growing population of young-onset patients and their FDRs. Furthermore, only 1 study has adopted a family-focused approach that delivers the intervention to both survivors and their FDRs [24]. This web-based intervention improved the comprehensiveness of SSE in hard-to-reach areas but did not affect CSE or sunscreen use [17].

### The Young Melanoma Family Facebook Intervention

To address this gap, we developed the Young Melanoma Family Facebook intervention to increase the performance of risk-reducing behaviors for young melanoma survivors and their FDRs. We selected a social media delivery platform for this intervention*.* Other than the web-based intervention evaluated by Bowen et al [17], prior behavioral interventions for FDRs and patients have been delivered via individual print materials or telephone counseling [18,22,23,25-27]. Our intervention work extends prior research by using a social media platform, Facebook, as the intervention delivery method. Given the ubiquitous use of social media, its potential for reach and communication presents a unique opportunity for health-promotion interventions. To date, most health communication research has used social media as a tool to improve knowledge and awareness of cancer risks [28], such as human papillomavirus awareness [29] and awareness of breast cancer risk [30]. Social media has also shown promise as a platform for behavior change interventions, including promising results in weight loss [31], physical activity [32], and tobacco use [33].

Facebook was a particularly suitable way to deliver intervention content to a group of young melanoma survivors and their FDRs for 2 reasons. First, this demographic has high rates of Facebook use [34], and most Facebook users are already connected to family members via Facebook. Our multiple-family Facebook group intervention approach includes both young survivors and their family members, and thus addresses the unique needs of the young adult survivor population, who have higher needs for peer and family support [35-37], prefer to interact with other young adult survivors [34], and prefer to obtain support from peer survivors rather than from other sources [38]. Our use of Facebook as an intervention platform is also a good match for this population’s preferences for information delivery, as they are frequent users of social media for health information [39]. Peer-to-peer and within- and between-family interactions have the potential to facilitate attitude and behavior change and set positive social norms for sun safety behavior.

The Young Melanoma Family Facebook intervention provides information, goal setting, and peer support to increase CSE, SSE, and sun protection behaviors for young-onset melanoma survivors and their FDRs. Content was guided by the Preventive Health Model [40-42] and the Theory of Normative Social Behavior [43,44]. The Preventive Health Model [41,42] is an integrative framework that includes the following constructs: risk, salience or coherence (benefits and barriers), and social and normative influences. The normative social influence construct is particularly important because of the family and group focus. The Theory of Normative Social Behavior proposes that normative influences are transmitted through interactions with family and peers about health behaviors, and these result in the internalization of norms. Facebook group participants may modify their own behavior and attitudes because of the norms about sun protection, CSE, and SSEs set by the intervention content and group members. If group members observe other members endorsing sun protection benefits and supporting regular self-examinations and CSE, normative changes may occur. Research on other group-based interventions supports this contention [45].

### The Melanoma Family Healthy Lifestyle Facebook Intervention

The literature on Facebook interventions is emerging, and there is no clear guidance on comparison groups. To select an appropriate comparison group, we reviewed the literature on the design of behavioral interventions [46]. This review suggested that there are pros and cons to all comparison conditions and that selection should be guided by the phase of the research. We selected a comparison condition that had the same method of delivery, same frequency of postings and group duration, and the same group composition. The key differences in the comparison intervention are as follows: (1) content is focused on healthy lifestyle (eg, nutrition, physical activity, and smoking cessation) and cancer survivorship and (2) content does not contain any material relevant to skin cancer, sun protection, SSEs, CSE, or tanning. This comparison condition offers a similar level of interactivity to that offered by the Young Melanoma Family Facebook intervention.

### Objectives

The purpose of this randomized clinical trial is to determine the efficacy of a Facebook intervention that provides information, goal setting, and peer support to increase CSE, SSE, and sun protection behaviors in young-onset melanoma survivors and their FDRs.

#### Primary Aim

The primary aim is to examine the efficacy of the Young Melanoma Family Facebook intervention versus a Melanoma Family Healthy Lifestyle Facebook intervention on CSE (primary outcome), SSE frequency and comprehensiveness, sun protection behaviors, and indoor tanning (secondary outcomes) of FDRs of young-onset melanoma survivors.

- Hypothesis: participants in the Young Melanoma Family Facebook intervention will report more CSE, more frequent and comprehensive SSEs, more sun protection behaviors, and less tanning at the 6-month follow-up compared with those in the Melanoma Family Healthy Lifestyle Facebook condition.

#### Secondary Aim 1

One secondary aim is to examine the efficacy of the Young Melanoma Family Facebook intervention on survivors’ SSE frequency and comprehensiveness and sun protection behaviors. Survivors’ behaviors are considered a secondary aim because the vast majority are compliant with CSEs. However, many survivors are not adherent to comprehensive SSE and sun protection behaviors; thus, we will focus on these outcomes.

- Hypothesis: survivor participants in the Young Melanoma Family Facebook intervention will report more frequent and comprehensive SSEs and higher sun protection at the 6-month follow-up compared with those in the Melanoma Family Healthy Lifestyle Facebook intervention condition.

#### Secondary Aim 2

Another secondary aim is to determine the mechanisms of intervention efficacy for both interventions’ impact on FDR and survivor outcomes.

- Hypothesis: based on the Theory of Normative Social Behavior [43] as well as the Preventive Health Model [40-42], we propose that the effects of the Young Melanoma Family Facebook intervention on FDR’s CSE, SSE, and sun protection behaviors and on survivors’ SSE and sun protection will be mediated by increased normative influences, family support and discussion, risk, benefits, barriers, self-efficacy, and use of change strategies.

#### Exploratory Aim

The exploratory aim is to evaluate the efficacy of the Melanoma Family Healthy Lifestyle Facebook intervention versus Young Melanoma Family Facebook intervention on perceived stress, physical activity, and healthy eating patterns among FDRs and survivors.

- Hypothesis: participants in the Melanoma Family Healthy Lifestyle Facebook intervention will report less stress, more physical activity, and healthier eating patterns at the 6-month follow-up compared with those in the Young Melanoma Family Facebook condition.

## Methods

### Ethics Approval

Study procedures were approved by the Rutgers University Institutional Review Board (IRB; Pro2019000158).

### Design

**Figure 1:**
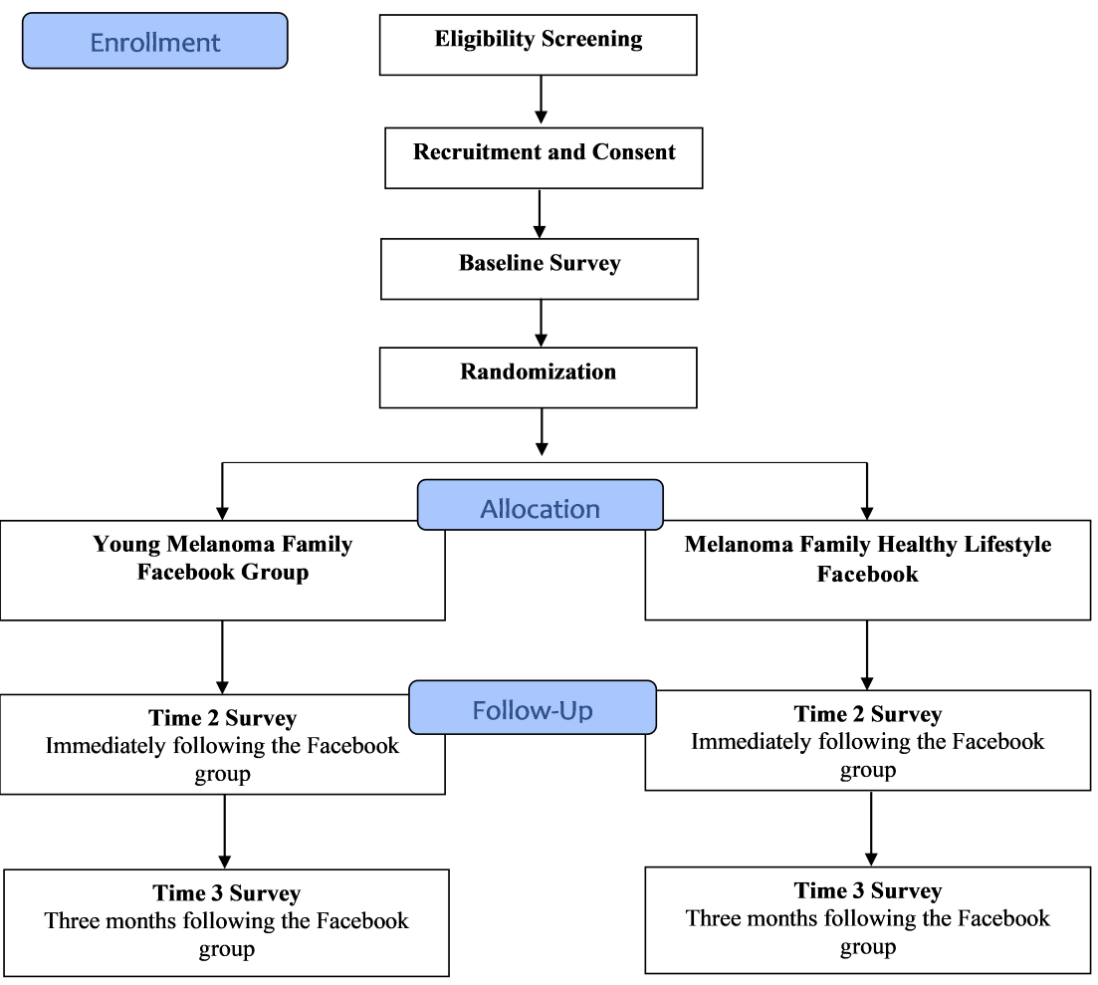
Participant flow.

This is a prospective, randomized controlled trial to evaluate the efficacy of the Young Melanoma Family Facebook intervention to increase engagement in CSE, SSE, and sun protection behaviors among young-onset melanoma survivors and their FDRs. Both the survivor and at least 1 FDR must consent and complete a baseline survey to be randomized. Eligible participants are randomly assigned to either the Young Melanoma Family Facebook intervention group or the Healthy Lifestyle Facebook intervention group and are asked to complete assessments at 3 time points: baseline, immediate postgroup intervention, and 3-month postgroup intervention. Members of the same family are assigned to the same condition. Figure 1 illustrates the overall design and participant flow of the study.

### Participants

Participants include melanoma survivors who have completed treatment within the last 5 years and their FDRs. We define treatment completion as the date of the last cancer treatment (eg, surgery). Spouses of survivors are considered eligible for the trial if 1 FDR consented to the study and if the survivor nominated their spouse for the study. This accommodation is created because of the family nature of this study. Spousal data are not included in the analyses.

### Recruitment

We use 2 different methods to recruit participants**:** state cancer registries and cancer centers. Survivors are recruited from the state cancer registries across the United States*.* Registry recruitment methods vary according to state laws. For some state registries, the registry confirms eligibility, approaches patients, and provides contact information to the main study site or sends contact information to the main study site. For other state registries, the registry identifies cases meeting the diagnosis time frame and current age and sends the list to the main site for recruitment. Eligible survivors are sent an electronic link to the informed consent. Once eligible, survivors provide the names, emails, and phone numbers of their parents, children aged >18 years, and full siblings. FDRs are contacted by telephone, and eligibility is confirmed. If eligible, FDRs are invited to participate. If interested, the electronic link to the consent and survey are provided by email. For both survivors and FDRs, calls are made 4 evenings per week and on weekends to ensure participants are reached. Up to 10 attempts are made to contact prospective participants by phone before they are deemed unable to locate.

To confirm eligibility before enrollment, the staff administer a series of screening questions to interested participants (Textbox 1). During eligibility screening, all survivors are asked to verify their date of birth, date of cancer diagnosis, internet access, and Facebook account status. All FDRs are asked to confirm their date of birth, internet access, and Facebook account status.

**Study eligibility.**

- Survivor
  Inclusion
    Diagnosed with stage 0-3 melanoma in the last 5 years
    Age at diagnosis 18-39 years
    Completed treatment at least 3 months previously
    At least 1 family member consents
  Exclusion
    Concurrent cancer diagnosis
    Cannot speak and read English
    Does not have access to computer, internet, and has a Facebook account
    Physical Activity Readiness Questionnaire [47]: this is a 7-item scale that used as a disclaimer to identify individuals who should consult a physician for medical clearance (if they mark yes to any of the 7 items) before engaging in physical activity in the event they are in the control group.

First-degree relative
  Inclusion
    Current age 18-80 years
    First-degree relative of survivor
    Survivor consents to the study
    Has only 1 first-degree relative with melanoma (survivor)
    Has not had a clinical skin exam in the past 3 years, has done SSE fewer than 3 times in the past year, OR has a sun protection habits average score ≤4 (“often”)
  Exclusion
    Does not have a personal history of melanoma
    Cannot speak and read English
    Does not have access to computer, internet, and has a Facebook account
    Physical Activity Readiness Questionnaire [47]: this is a 7-item scale that used as a disclaimer to identify individuals who should consult a physician for medical clearance before engaging in physical activity in the event they are in the control group.

### Consent, Enrollment, Randomization, and Follow-up Survey Procedures

Once the prospective participant’s eligibility is confirmed, informed consent is obtained using an electronic consent form hosted on DatStat. Participants are informed of the study requirements, the potential risks and benefits of study participation, and a breakdown of study compensation. After obtaining informed consent and baseline surveys, participants are assigned a study ID number. Both the survivor and at least 1 FDR must consent and complete a baseline survey to be randomized. Thus, randomization occurs only after both the survivor and 1 FDR complete these tasks. Participants are randomized to either the Young Melanoma Family Facebook Group or the Melanoma Family Healthy Lifestyle Facebook Group. The randomization scheme was developed by the study statistician. Clusters of participants (survivor + FDRs) are randomly assigned to conditions in blocks of 50 FDRs to achieve our target group size of approximately 25 survivors and 25 FDRs assigned to each group (Young Melanoma and Healthy Lifestyle). Members of the same family are assigned to the same condition. If additional FDRs join the study, they are assigned to the same condition. The team’s project coordinator allocates participants to their randomized condition and notifies them of the study assignment via email.

Upon family randomization, participants are provided with the Facebook name of the intervention account that they have been randomly assigned to and asked to “friend” that respective account. Once participants “friend” the Facebook group account, the staff member posts a link into the intervention Facebook group account that participants use to authorize the Grytics software to capture their engagement data [48]. When a participant clicks the opt-in button, it redirects the participant to their own Facebook settings and provides more details about the information the app will be collecting (Facebook name, profile picture, posts, comments, and reactions). The participants have the option to indicate which of their Facebook groups the Grytics software can see by clicking “choose what you allow.” When the participant accepts the invite, their request to join the group stays in “pending” status until the group administrator approves all members on day 1 of the group. The study staff contact those who do not “friend” the Facebook accounts.

After group completion, the participants are sent a link for the first follow-up survey and intervention evaluation, which is completed on the web. Three months after group completion, the participants are sent a link for the second follow-up survey, which is completed on the web.

### Intervention Conditions

#### Young Melanoma Family Facebook Group

Participants are asked to join a secret Facebook group in which membership and content is only viewable by invited group members. The participants’ group membership and activities are not publicly viewable to external Facebook users. The groups run for 12 weeks and contain up to 54 members (25 survivors and their FDRs). Posts are made twice a day for the first 8 weeks of the group, which is the frequency suggested by social media marketers to engage participants without overburdening them [49]. For weeks 9 to 12, a total of 4 to 6 posts are made each week. Each week is organized around one of the following topics: melanoma risk, physician skin examination, skin self-check, and sun protection habits. The first 4 weeks are organized according to the following topics: risk factors, CSE, skin self-check, and sun protection habits. Behavior change strategies such as goal setting and increasing support for CSE, SSE, or sun protection are woven into posts. During weeks 5 to 8, content is expanded for each topic, special topics are addressed, and news and stories about skin cancer are presented. Messages reinforce regular sun protection, CSE, SSE (eg, mole map pictures), and group discussions. The surgical oncologist and content expert on skin cancer (AB) will reply to the “Ask the Expert” postings in the Young Melanoma Family Group for questions that are not already included in the Question and Answer Library (Textbox 2).

**Figure 2:**
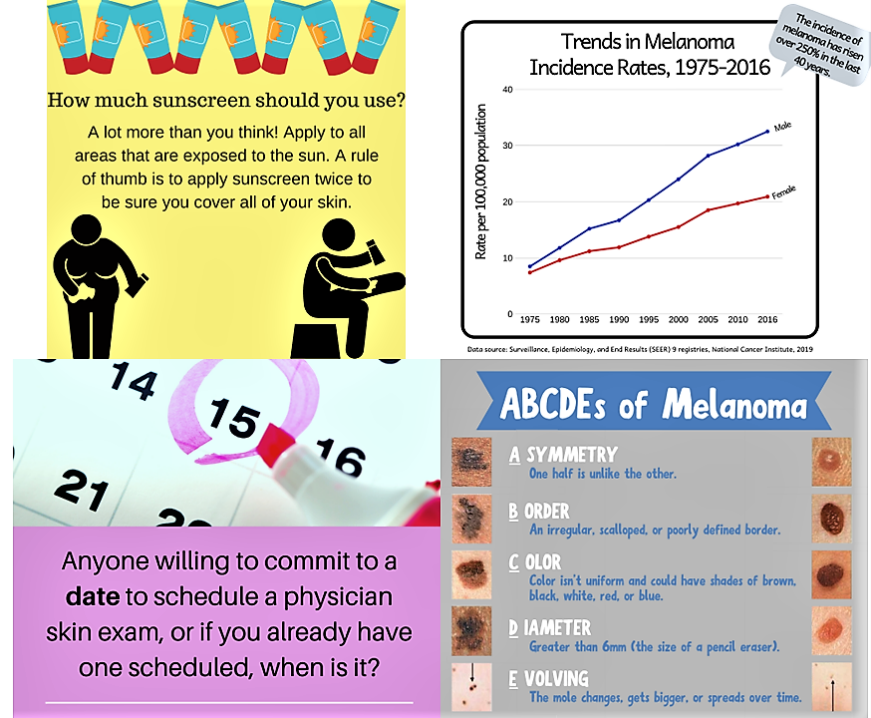
Sample Facebook posts for the Young Melanoma Family Facebook Group.

Sample posts are shown in Figure 2. Engagement is enhanced by engaging with humorous pictures, links to educational materials, polls, questions (“What is the ultraviolet light index in your town today?” “What is your favorite brand of sunscreen?”), memes, and holiday greetings. Each group ends at 12 weeks, at which time participants are no longer able to post. However, participants will be able to review the previous 12 weeks of postings.

**Young melanoma Facebook group content.**

- Risk: information about elevated risk among relatives, information about skin cancer incidence among young adults, phenotypic risk factors, association between UV light exposure and skin cancer, Fitzpatrick skin type quiz.
- Clinical skin exams: information about clinical skin examination recommendations for persons at higher risk for melanoma, information about what is involved in a clinical skin examination, benefits of having an examination, barriers to having an examination, and opportunities to ask questions to a skin cancer expert.
- Skin self-check: professional recommendations regarding skin self-examination, how to prepare for and perform a skin self-check, how to recognize a suspicious growth, why skin self-examination is important in melanoma risk reduction, how to find a dermatologist, benefits of skin self-examination, barriers to skin self-examination, goal setting for skin self-examination, finding assistance in conducting a skin self-examination, what to do if something suspicious is found.
- Sun protection habits: sun safe behaviors, what sun protection factor means, importance of clothing that covers the body, why wide-brim hats are more protective, common barriers to sun safe behaviors and ways to address barriers.
- General engagement: fun and engaging posts such as holiday memes.

#### Melanoma Family Healthy Lifestyle Facebook Group

**Figure 3:**
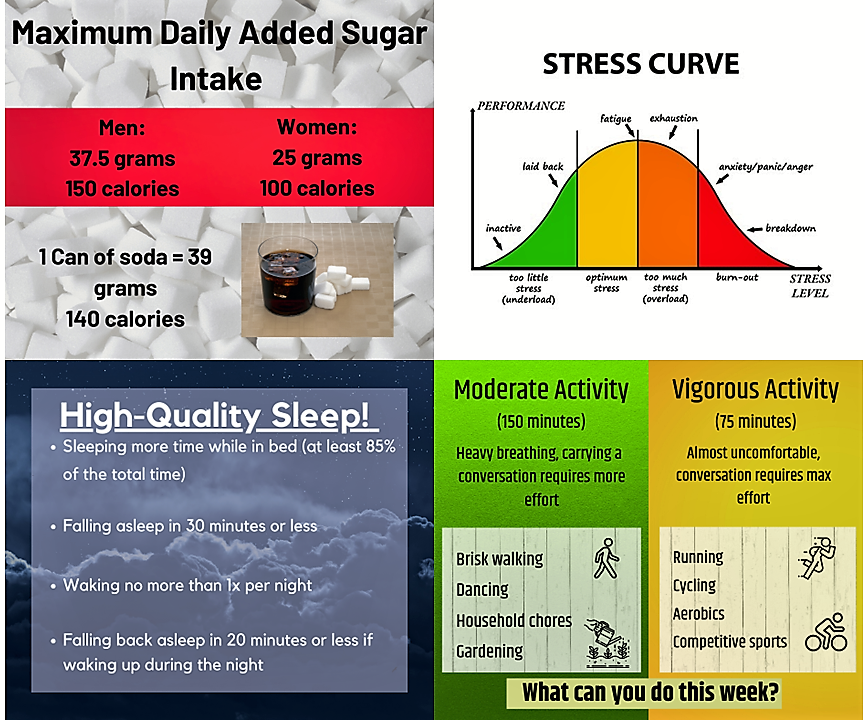
Sample Facebook posts for the Melanoma Family Healthy Lifestyle Facebook Group.

The procedures for joining the group, Facebook privacy settings, moderation guidelines, frequency of posts, group duration, and access after the group ends are the same as for the Young Melanoma Family Facebook Group. Each week is organized around one of the following topics: nutrition, physical activity, sleep, and stress. Sample posts are shown in Figure 3. Engagement is enhanced by including humorous memes, polls, conversation starters, and seasonal greetings (Textbox 3).

**Melanoma Family Healthy Lifestyle Facebook Group content.**

- Nutrition: content focuses on maintaining a healthy diet, benefits of a healthy diet, barriers to maintaining a healthy diet, and ways of improving dietary practices.
- Physical activity: content focuses on recommended levels of physical activity, types of physical activity, benefits of regular physical activity, barriers to achieving recommended levels of physical activity, and ways of improving physical activity levels.
- Sleep: content focuses on recommended number of hours of sleep, symptoms of sleep deficits, common reasons why people do not sleep well, benefits of getting a good night’s rest, and strategies to improve sleep habits.
- Stress: content focuses on signs and symptoms of stress, benefits of reducing stress, reasons why people have problems managing stress, and strategies to reduce stress.
- General engagement: content includes fun and engaging posts, such as memes about exercise

#### Moderator Activities

The moderator logs in 2 to 3 times a day to review the activity and facilitate engagement by “liking” participants’ comments and replying with supportive responses. Because Facebook norms are for brief and frequent interactions, moderator responses will be brief, but engage participants in discussions, reinforce engagement (eg, using a “like” reaction), and answer questions. The study collaborator, SP, reviews Facebook activity on a regular basis.

### Facebook Group Privacy and Appropriate Behavior in Groups

To ensure the privacy and safety of participants in the Facebook groups, the study team takes the following measures. First, the study’s informed consent documentation process includes a description of the privacy settings for Facebook groups. Second, during a phone contact with each participant before the group’s initiation, the research staff describes what a “secret” Facebook group means in terms of privacy (eg, group and its contents are not visible to the public), the privacy limitations for a “secret” Facebook group (eg, cannot guarantee that other participants will not share content posted with others), and the confidentiality rules of the group. Third, if the study team learns that a participant has breached confidentiality in any way, we contact the participant to discuss confidentiality. Any breach in confidentiality is reported as a study adverse event and is reviewed by the study team on a bimonthly basis. In terms of appropriate posts by members, the moderator notifies the principal investigator (PI; SM) if unanticipated, distressing, or inappropriate comments are posted in the Facebook group. The study team will discuss the appropriate response to the issue and will provide guidance to the moderator on how best to proceed.

### Enrollment Timeline

Enrollment began in January 2019. Recruitment is ongoing, and we anticipate completing recruitment in November 2023.

### Measures and Assessment Periods

Participants complete electronic surveys via DatStat at 3 time points. Each self-report survey takes approximately 30 minutes to complete. The baseline survey is completed between 1 and 2 months before the initiation of a new Facebook group. The second survey is completed immediately after the group ends (approximately 3 months after group initiation). The third survey is completed 3 months after the group ends. To facilitate completion, research staff members reach out to participants who do not return a weekly survey. The data management program, DatStat, is programmed to send 3 reminder emails. Staff members complete a minimum of 1 phone call for incomplete surveys.

### Primary and Secondary Outcome Measures

#### Clinical Skin Examination

The participants are asked about the last time a physician checked any part of their body for early signs of skin cancer (within the last month, 1-6 months ago, 7-12 months ago, >1 year ago, and never)*.* CSE is defined as whether the participant had undergone such an examination in the previous year (yes or no). We expect that close to 100% of survivors will have had a CSE in the past year; thus, analyses of this outcome will focus on FDRs.

A randomly selected subset of 50% of the reported CSEs are confirmed by the physician or facility completing the CSE. The participants are asked to complete an electronic Health Insurance Portability and Accountability Act medical release form to provide the name and contact information of the provider or medical facility to verify the procedure and the date on which it was performed. The study staff member contacts the provider through either email or fax and requests verification of the procedure and the procedure date.

#### Skin Self-examination

Participants are asked whether they had checked any part of their body for early signs of skin cancer in the last 12 months. Participants who report that they checked their skin at least once indicate the number of times they checked their skin in the last 12 months, the last time they checked their body, and the specific areas that they thoroughly examined during their last skin check. Two outcomes are used: SSE performance in the past year (*yes or no*) and SSE comprehensiveness (the number of body parts [out of 15] checked in the last SSE). Note that because SSE (yes or no) overlaps with the number of body parts such that everyone with a “no” will have 0 body parts, we limit the analysis of the number of body parts to scores ranging from 1 to 15, omitting zeros from the analysis.

#### Sun Protection Behaviors

Participants rate how often they engaged in 6 behaviors in the last month: stay in the shade, wear a long sleeve shirt, wear long pants or cover legs, wear a wide-brimmed hat, wear sunglasses, and wear sunscreen with a sun protection factor ≥15 (1=*never* to 5=*always*) [50].

#### Indoor Tanning

Participants rate how many times they engaged in indoor tanning (tanning bed, sun lamp, and tanning booth) in the last 3 months.

### Exploratory Outcome Measures

#### Perceived Stress

The Perceived Stress Scale-4 [51], designed for use in community populations, is a widely used psychological instrument for measuring the perception of stress. It is a measure of the degree to which situations in one’s life are appraised as stressful in the previous month (“How often have you felt that difficulties were piling up so high than you could not overcome them?” 1=*never* to 5=*very often*).

#### Physical Activity

The Godin Leisure-Time Exercise Questionnaire [52] is a 4-item widely used self-report measure for describing patterns of vigorous and moderate physical activity during the last 7 days (eg, “How many days did you do vigorous physical activities like heavy lifting, aerobics, or fast bicycling?” *number of days/week with no vigorous activities*).

#### Healthy Dietary Practices

Four items assess how many days per week, over the last 7 days, that the participant has consumed fast food (*<1, 1-3, or ≥4*), daily servings of fruits and vegetables (≥*5, 3-4, or ≤2*), daily servings of nondietary soft drinks (*<1, 1-2, or ≥3*), and times per week desserts or other sweets are consumed (≤*1, 2-3, or ≥4*) [53].

### Mediator Variables

#### Perceived Risk for Skin Cancer and Recurrent Melanoma

The perceived risk of melanoma is assessed using 4 items [54]. Sample items are “How would you rate your chances of developing melanoma as compared with other people with a similar family history of melanoma?” (1=*much higher than other people*, 5=*much lower than other people*) and “I may get a skin cancer if I don’t protect my skin from the sun” (1=*strongly disagree*, 5=*strongly agree*).

#### Benefits

Six items assess benefits of engaging in sun protective behaviors (1=*strongly disagree* to 5=*strongly agree*) [55,56]. Nine items assess the benefits of engaging in CSE [55,57,58]. Ten items assess the benefits of engaging in SSE (1=*strongly disagree* to 5=*strongly agree*) [55,57,58]. An average is computed, and higher scores indicate more benefits.

#### Barriers

Fifteen items assess common barriers to using sunscreen and wearing sun protective clothing (1=*strongly disagree* to 5=*strongly agree)* [55,56]*.* Seven items assess common barriers to having a CSE, and 8 items measure common barriers to having an SSE [55,57,58]. The averages are computed for each scale, and higher scores indicate more barriers.

#### Self-efficacy

Eight items measure confidence in using sunscreen in various settings: 1=*not at all confident,* 5=*very confident* [55,56]. Eight items assess confidence in being able to conduct SSE [57,58].

#### Family Norms

Perceived attitudes and practices of other family members regarding tanning and sun protection are assessed using 9 items (1=*strongly disagree*, 5=*strongly agree*) [59], perceived attitudes and practices of other family members regarding CSE with 5 items [60], and perceived attitudes and practices of other family members regarding SSE with 6 items [60]. Higher scores indicate stronger norms.

#### Family Benefits for Engaging in Risk-Reduction Practices

Nine items measure perceived benefits to one’s family if the individual engages in risk-reducing practices, as well as benefits for the individual if their family engages in risk-reducing practices*.* Items were adapted from our prior work on the relationship between the benefits of cancer screening [61] and sun protection [62]. Higher scores indicate more benefits for the family.

#### Family Discussion

The frequency of discussions regarding sun protection with the participant’s sibling, parent, spouse, and children in the past month are reported in 4 items (eg, “How often did you discuss sun protection with your siblings, parents, children, or spouse?” *never=*0*, once=*1*, twice=*2*,* or *more than two times=*3) [59,61]. A sum of these items will be computed. However, because a participant may not have one or more of these family members (eg, they are not married or they do not have children), the family discussion variable will be computed by dividing the sum score by the number of available family member types. For example, a person with a parent, spouse, and child could have a maximum score of 9 on the sum measure. Therefore, the family discussion variable is computed by dividing the sum by 9 and multiplying by 100 to create a score that could range from 0 to 100. This approach was used in our prior work [63].

Three items assess the frequency of discussions about CSE and 3 items assess the frequency of discussions about SSE with the participant’s siblings, parents, and spouses (ie, participants were not asked whether they discuss CSE or SSE with their child; *never*=0*, once*=1*, twice=2,* or *more than two times=3)*. As with sun protection, the family discussion variables for SSE and CSE will be computed by dividing the sum of the frequency of discussions by the number of available family member types to create a score that could range from 0 to 100. A score of 100 indicates that the person has discussed CSE (or SSE) at least 2 times with all available family members. Note that although we know that the person discussed CSE and SSE with at least 1 sibling, for example, we do not know if they discussed it with every sibling they have.

#### Action Planning

On the basis of prior work [59,64], 4 items were created to assess whether the participant made a detailed plan regarding when, where, and how to engage in sun protection behaviors and what to do if anything interferes. Using 4 items, we ask whether the participant made a detailed plan regarding when, where, and how to have a CSE and what to do if something interferes; 4 items assess whether the participant made a detailed plan regarding when, where, and how to do an SSE and what to do if something interferes (1=*strongly disagree,* 5=*strongly agree*). Higher scores indicate more planning.

#### Facebook Group Social Network Influence

##### Overview

Social network analysis (SNA) and content analysis of participant posts, comments, and reactions will be used to understand the mechanisms through which interactions in Facebook groups result in behavior and attitude change. This measures how much participants engaged with each other and whether these peer-to-peer engagements are predictive of the outcomes. Social influence is a function of three contextual factors: (1) traits of members of the reference group, (2) content of Facebook messages, and (3) social network exposure. We use SNA to derive individual, dyadic, and group-level measures to examine the role of the social structural context on normative influence [65-67]. Ties will be measured using the following Facebook data: (1) reactions (likes); (2) comments to member posts and comments; and (3) existing familial relationships. Tie measures will be combined to measure tie strength (0=*no tie*, minimum of 1=*tie*, strength=*sum of all ties across the weeks the group is running*; Textbox 4).

In addition, comments will be content analyzed using a codebook. The codebook will be developed through an iterative process that first started with a literature review to identify common themes that emerge in social media health intervention studies [72-77]. Examples of content-type themes from our review of the literature include “awareness,” “support,” “promotion,” “information seeking,” and “support seeking,” among others. In addition to content-type themes, we will use themes that characterize content expression. This regards how comments are expressed and include “supportive,” “humorous,” “evaluative,” and sentiment (positive, negative, and neutral affect). More themes will be identified through an initial reading of a randomly selected subset of 200 comments, across the 6 intervention waves. This open reading process will identify recurring themes [78] for an initial codebook. These codes will be applied to another randomly selected subset of 200 comments by a team of 2 trained coders, to establish an acceptable interrater reliability score (Cohen κ of at least 0.70). In discussions between coders and research team leads, the codebook will be refined by adding any additional emergent themes and merging similar themes into broader categories. This refined codebook will then be applied to a final subset of 200 comments to ensure improved interrater reliability among the team’s coders. The coding team will then code the entire data set of comments. Comments could be coded for multiple themes. Each comment will include information on the number of “likes” and comments that it received [74]. The content analysis will be used in two ways: (1) determine theme frequency for each group and (2) codes (presence=1, absence=0) will be aggregated across all comments made by each group member (excluding project team). Thus, we will have summaries of each code for each participant, which will be used, along with network measures, to assess participants’ levels of engagement, helpfulness (eg, number of informational comments), supportiveness (eg, number of supportive comments), and any other themes our team deems to be relevant for influence at the codebook development stage.

**Facebook-based social network context measures.**

- Individual traits
  Centrality: assess influence based on network position [68]
  Survivor vs first-degree relative: as melanoma survivors, Facebook posts about risk or prevention are assumed to be influential
- Message content
  Posts: information, stance, and sentiment communicating attitude about intervention and participant-posted content [69]
- Social Network exposure
  Dyadic: ties between group members Feedback: likes and comments that communicate support, agreement, and encouragement [67] Reciprocity: likes and comments in response to feedback [70]
  Group-level: group structure Subgroups: Subgroup formation based on high frequency interaction and consistent interests [71]

##### Facebook Engagement

###### General Use of and Connectedness With Facebook

Facebook use will be measured with 3 items (eg, log-ins per week, membership in private groups, or in public groups). Participants will complete the Facebook Intensity Scale [79], which consists of a 5-item experiential measure of Facebook use separate from behavioral measures of frequency and duration, incorporating emotional connectedness to the site and its integration into individuals’ daily activities (eg, “I feel I am part of the Facebook community”). Ratings range from 1=*strongly disagree,* 5=*strongly agree*, and higher scores indicate more connections with Facebook.

###### Facebook Group Engagement

Facebook data will be extracted to assess behavioral engagement using Grytics extraction software to capture views, likes, and comments on posts. We will calculate descriptive information for each post, group cohort, and individual participant. Participant engagement (ie, reactions, replies, poll votes, and posts) will be analyzed as a predictor of response to treatment in the analyses.

#### Intervention Evaluation

Participants in both intervention conditions rate 18 items based on helpfulness, value, relevance, and accuracy of the materials posted, as well as aspects of group participation, such as comfort with participation, feeling connected with, actively involved with, enjoying expressing opinions, and reading posts (1=*not at all*, 7=*extremely*).

### Covariate Measures

#### Demographic Information

Participants report their age, sex, race or ethnicity, education level, insurance status (*yes or no*), and whether they see a primary care provider at least once a year (*yes or no*).

#### Objective Risk Factors

Skin color, natural hair color (higher score: blonde or red hair), eye color (higher score: blue or green eyes), history of sunburns, skin reactivity to the sun (the degree to which the individual burns or tans when exposed to the sun, tanning ability, and depth of tan), freckling, and sensitivity to the sun (very sensitive to very resistant) are assessed.

#### Medical Factors (Survivor Only)

Data on months since melanoma surgery, stage of disease, and location of cancer are collected from medical charts.

#### Stakeholder Interviews

To inform future potential dissemination and implementation of the Young Melanoma Family Facebook Group intervention, we will conduct semistructured key informant interviews with three types of stakeholders: (1) 5 providers (cutaneous oncologists, dermatologists); (2) 5 cancer advocacy group leaders (eg, Stupid Cancer); and (3) 30 study participants (survivors and FDRs). Study participants will be purposively selected from the Young Melanoma Family Facebook Group condition, including approximately 15 survivors and 15 FDRs, and representing variation in participation in the intervention and scores on the 18 intervention evaluation items. Interviews (45-60 minutes in length) will be conducted using videoconferencing software and audio recorded with participant’s permission. Interview participants will be compensated US $50.

Interview guides will be tailored to each of the 3 stakeholder types to assess multiple perspectives on the acceptability, feasibility, and appropriateness of the intervention. Providers and cancer advocacy group leaders will be introduced to the intervention at the beginning of their interviews. Interview questions and probes (as appropriate for each stakeholder group) will focus on (1) current approaches and practices to promote CSE, SSE, and sun protection; (2) perceived benefits and shortcomings of the Young Melanoma Family Facebook intervention; (3) actual and potential barriers to its use and delivery; (4) possible dissemination channels [80] to reach survivors and their families (eg, communication modes, provider recommendation, and social marketing); (5) pros and cons of suggested dissemination channels and strategies; (6) the “fit” of the intervention with the preferences of survivors and FDRs and the missions and objectives of providers and organizations [81]; and (7) resources, infrastructure, and other factors needed to implement and sustain the intervention outside of the research context.

## Results

### Approach to Missing Data

We will use an intent-to-treat approach to the analyses of primary and secondary outcomes. Our approach to recruitment and retention minimizes missing data by contacting participants who do not complete sections of the survey and by incentivizing participation. Initial analyses will examine the characteristics of noncompleters. As recommended by Lang and Little [82], multiple imputations (using 50 imputed samples) will be used to impute missing values (Table 1).

**Table 1:** Study measures.

Measure		Screening	Baseline	Time 2	Time 3
**Covariates**					
	Date of birth	✓			
	Sex		✓		
	Survivor date of diagnosis^a^		✓		
	Race and ethnicity		✓		
	Education		✓		
	Insurance status		✓		
	Regular primary care		✓		
	Phenotypic risk		✓		
**Outcomes**					
	Clinical skin examination	✓	✓	✓	✓
	Skin self-examination	✓	✓	✓	✓
	Sun protection behaviors	✓	✓	✓	✓
	Perceived stress		✓	✓	✓
	Physical activity		✓	✓	✓
	Dietary patterns		✓	✓	✓
	Indoor tanning		✓	✓	✓
**Mediators**					
	Risk		✓	✓	✓
	Benefits		✓	✓	✓
	Barriers		✓	✓	✓
	Self-efficacy		✓	✓	✓
	Family norms		✓	✓	✓
	Family benefits		✓	✓	✓
	Family discussion		✓	✓	✓
	Action planning		✓	✓	✓
**Facebook intervention variables**					
	Facebook engagement				✓
	Connectedness with Facebook	✓			
	Facebook social influence				✓
	Treatment evaluation			✓	
**Qualitative data**					
	Stakeholder interviews				✓

### Approach to Data Management

Although survivors are asked specifically to nominate parents or siblings as FDRs, experience indicates that some survivors will nominate spouses who are not FDRs. If this occurs, the spouse’s data will be excluded from the analyses. A second complication in this area is when children are nominated. Although children are FDRs, our focus on young-onset melanoma survivors will likely result in a sample of survivors who rarely have children who are old enough to participate in the study. Given the distinct roles played by the different FDRs (ie, parental relationships and influence that likely differ from sibling relationships and influence), children will be included in analyses as FDRs only if they represent at least 5% of the FDRs in the study, so that the differences among parents, siblings, and children can be examined. Finally, after excluding spouses (and possibly children if the sample of children FDRs is small), there may be survivors with no valid FDRs in the study. Those survivors will be removed from analyses.

The treatment of covariates warrants further investigation. Possible covariates include demographic variables, objective risk variables, and medical variables for the survivor. To determine which covariates should be included in the analyses, preliminary analyses will be conducted in which each outcome is predicted by the full set of qualifying covariates. Only those covariates that significantly predict at least 1 outcome will be included in the analyses. Once the set of covariates is determined in this manner, the same set of covariates will be included in all analyses. Any covariates that are limited in their variation will be excluded from the analyses. Consider race or ethnicity as an example: rates of melanoma are strongly tied to race and ethnicity, with the highest rates for White, non-Hispanic individuals. If the distribution of race or ethnicity in the sample is limited (ie, 90% or more of the sample is White, non-Hispanic), race or ethnicity will not be included in the analyses. In addition to race or ethnicity, we anticipate that insurance status, visits to a primary care provider, and stage at diagnosis for the survivor may also show low variation; if so, they will be excluded from the analyses.

Survivors and FDR ages also present potential problems as covariates. The analysis described in subsequent sections systematically tests whether there are differences in outcomes as a function of role (survivor, parent, or sibling), sex, and their interaction. The interaction tests differences for mothers versus fathers, sisters versus brothers, and male survivors versus female survivors. Participant age will likely correlate strongly with the role variable (ie, parents will be relatively older than siblings and survivors) and, if so, age will not be included as a covariate in analyses to avoid multicollinearity.

### Primary Aim, Secondary Aim 1, and Exploratory Aim Analysis Plan

The primary aim is to examine the efficacy of the Young Melanoma Family Facebook intervention on CSE, SSE frequency, comprehensiveness, and sun protection behaviors of FDRs after completion of the intervention, and the first secondary aim is to examine the efficacy of the intervention on *survivors’* SSE frequency and comprehensiveness and sun protection behaviors after completion of the intervention. These 2 aims (*primary aim and secondary aim 1*) will be addressed together using multilevel modeling (MLM) because the data from FDRs and survivors are assumed to be interdependent. The *exploratory aim* of testing whether the intervention is effective in reducing perceived stress and improving other outcomes such as physical activity will also use this approach. MLMs with the following attributes will be used to address the primary aim and secondary aim 1:

- Family is the upper-level sampling unit and individual (FDR or survivor) is the lower-level unit.
- Fixed effects variables include condition (Young Melanoma versus Melanoma Family Healthy Lifestyle Facebook), respondent role (parent, sibling, or survivor), respondent biological sex, all 2- and 3-way interactions, and the set of covariates, including the individual’s baseline score on the outcome.
- Random intercepts will model interdependence within families.
- Models predicting dichotomous outcomes (CSE and SSE yes or no, any indoor tanning) will be estimated using binary logistic multilevel models.
- A standard multilevel analysis will be conducted to predict the SSE comprehensiveness of sun protection behavior, perceived stress, physical activity, etc, using the same fixed- and random-effect parameters.
- Exploratory analyses of moderators of intervention efficacy will be conducted using this MLM approach by including each moderator (eg, melanoma risk factors and month of the year enrolled) as an additional fixed effect factor in the model.

### Power Calculations

Power analyses were computed assuming a total of 1154 participants (577 survivors and 577 FDRs). Outcomes were assumed to correlate within families with *r*=0.5. Given those values, the effective sample size taking clustering into account is a total of 773. Using a .05 significance level and 90% power, this sample size is sufficient to detect small effects for CSE (Cohen *d*>0.12). For SSE, the effect size was an overall increase in SSE in the Young Melanoma Family Facebook condition of 16.1% across FDRs and survivors. Using pretest-posttest pilot data, we found Cohen *d*=0.14 for sun protection behaviors across FDRs and survivors.

### Secondary Aim 2: Analyses

This aim evaluates hypothesized mediators of the expected association between the intervention and outcomes (FDR engagement in CSE, FDR, and survivor engagement in self-examinations and sun protection behaviors). Mediators that are outcome-specific (eg, benefits of engaging in CSE, SSE, and sun protection) include benefits, barriers, family norms, family discussions, and action planning. Mediators that are not outcome-specific include sunscreen self-efficacy and family benefits for risk reduction.

**Figure 4:**
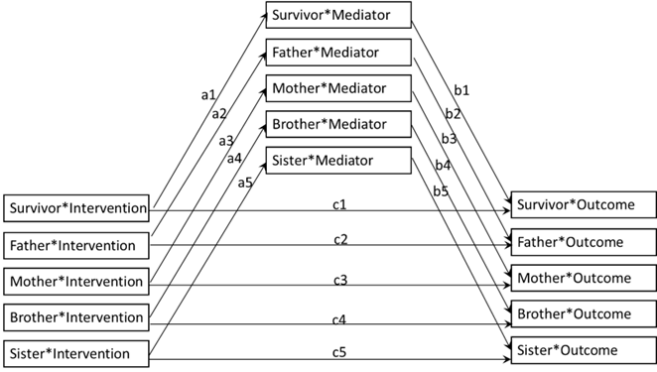
Mediation model.

Mediation analyses will assess whether the effects of the intervention on outcomes are mediated by changes in family and normative factors (family discussion, family norms, and family benefits) and individual attitudes (eg, risk, benefits, barriers, self-efficacy, and action planning). MLM will be used to estimate the key mediation paths using 4-component steps [83]. These MLMs will include dummy-coded variables to denote the combination of biological sex and participant role (eg, mother or father). Each of these dummy variables will also interact with the treatment condition, and the overall intercept in this type of simple slope model is suppressed. To assess mediation (Figure 4), these predictors are included in a model that predicts the proposed mediator, and the coefficients from the interaction between the dummy variables and condition estimate the “a” path for each role. To assess the “b” paths, the interactions between the dummy variables and mediator are added to the model, and the outcome (CSE, SSE, or sun protection) is predicted. The contribution of the mediated effect will be assessed directly by using the approach recommended by Kenny et al [84] for multilevel models. Specifically, the product of the 2 pieces of the mediating path (ie, “a*b” from intervention group assignment to mediating variable and from mediating variable to outcome) will be calculated. Bootstrap procedures will be used to test the significance of the mediating pathway.

To inform our interpretation of outcomes, we will examine social influence dynamics in both conditions. We conceptualize social influence as a function of three contextual factors: (1) traits of members of reference group; (2) content of messages; and (3) social network exposure. We will use SNA and content analysis of posts to understand the mechanisms through which interaction in Facebook groups results in behavior and attitude change. We use SNA to derive individual, dyadic, and group-level measures to examine the role of social structural context on normative influence. Ties will be measured as (1) reactions (likes); (2) comments to member posts; and (3) existing familial relationships. Tie measures will be combined to measure tie strength (0=no tie, minimum of 1=tie, strength=sum of all ties across the weeks the group is running). Once ties are established, SNA software Ucinet [85,86] will be used to compute network measures. We will derive variables from the content analysis of comments posted to the group that, along with network variables, will assess Facebook group members’ influence—both individual members and in terms of broader group dynamics (eg, multiple members expressing consensus on a particular topic). Information, support, stance, and sentiment are some of the content-related characteristics that can drive influence. Other relevant variables may arise through the content analysis, as described earlier.

### Secondary Aim 2: Sample Size and Power

Sample size calculations for mediated effects are based on simulation studies that model the 2 pieces of the mediating path (study arm to mediator; mediator to outcome) [87]. Assuming 583 survivors with 22% dropout, there is greater than 80% power to detect paths with regression coefficients of 0.33 (small-to-moderate effects) [88]. Assuming 577 FDRs with 22% dropout, there is 80% power to detect paths with regression coefficients between 0.20 and 0.23 (small effect sizes). With smaller intraclass coefficients, the effective size could be closer to 0.20.

### Qualitative Analyses of Stakeholder Interviews

The audio recordings of key informant interviews will be transcribed verbatim. Transcript data and notes will be imported into ATLAS.ti software to facilitate analysis. Both deductive and inductive coding processes will be used [89]. Deductive directed content analysis will allow specification of constructs of interest a priori for coding (eg, acceptability, feasibility, appropriateness, and barriers), whereas an inductive coding process will allow development of additional codes identified through immersion in the transcripts. All qualitative coding procedures will follow rigorous procedures to ensure consistency and a close connection among the data and identified codes and categories [90] (eg, independent initial code development by multiple qualitatively trained research staff, iterative refinement of the codebook based on team meetings to review codes and resolve discrepancies, 6 randomly selected transcripts double-coded with the final codebook). Once coding is complete, the team will develop a summative grid of themes across and within stakeholder groups (providers, cancer advocacy group leaders, and survivors and FDRs), yielding multiperspective insights into multilevel dissemination and implementation strategies needed for future scale-up, dissemination, and implementation. Reporting of qualitative results will follow the Consolidated Criteria for Reporting Qualitative Research [91].

### Data Safety and Monitoring

An adverse event in this study is defined as follows: (1) a participant dying between baseline and follow-up (not a study-related adverse event), (2) a participant reporting distress associated with study participation or (3) if the participant has breached confidentiality in the Facebook group (eg, sharing a group member’s post that contains their name outside of the Facebook group). Participants reporting distress will be contacted by the site’s PI. During this telephone contact, the causes and degree of distress will be discussed. If the site PI feels there is a need for referral of the participant for psychological intervention, they will identify providers near the participant and provide the referral information. Participants breaching confidentiality will be contacted to let them know that they cannot share posts outside of the group. All adverse events will be documented by the study staff and reviewed on a monthly basis by the study PI and the project coordinator and reported to the safety officer. The IRB and Rutgers Cancer Institute of New Jersey’s Protocol Monitoring Committee reviews all adverse events. The National Institutes of Health and Rutgers IRB will be notified immediately in the event of serious adverse reactions or any unanticipated (not mentioned in the consent form) occurrence of physical or psychological harm or unexpected threat to privacy (eg, lost records) or safety of the participants.

## Discussion

### Principal Findings

Once the analyses are completed, we anticipate that FDR participants in the Young Melanoma Family Facebook intervention will report more CSE, more frequent and comprehensive SSEs, and more sun protection behaviors at the 6-month follow-up compared with those in the Melanoma Family Healthy Lifestyle Facebook condition. We also predict that survivor participants in the Young Melanoma Family Facebook intervention will report more frequent and comprehensive SSEs and higher sun protection at the 6-month follow-up compared with survivor participants in the Melanoma Family Healthy Lifestyle Facebook intervention condition. Indoor tanning is an exploratory outcome, and we propose that participants in the Young Melanoma Family Facebook intervention will report lower engagement in indoor tanning at the 6-month follow-up than participants in the Healthy Lifestyle Facebook intervention.

In terms of moderators and mediators, we expect that younger participants, those who engage more in the intervention and are heavier Facebook users will respond more favorably to the Young Melanoma Family Facebook condition. We expect that the effects of the Young Melanoma Family Facebook intervention on FDR’s CSE, SSE, and sun protection behaviors and on survivors’ SSE and sun protection will be mediated family support and discussion, risk, benefits, barriers, self-efficacy, use of change strategies, and by increased normative influences. The latter will be measured through variables derived from the SNA (eg, social network variables like centrality, reciprocity, and subgroup formation) and from the content analysis (eg, member comment informativeness, supportiveness, and sentiment). Finally, we expect that participants (FDRs and survivors) in the Melanoma Family Healthy Lifestyle Facebook intervention will report less stress, more physical activity, and more healthy eating patterns at the 6-month follow-up compared with those in the Young Melanoma Family Facebook condition.

### Limitations

The results of this trial will be evaluated in the context of its limitations. For example, there are challenges with the Facebook delivery platform, recruitment of family members of survivors, and reliance on self-report measures of SSE and sun protection. First, Facebook posts tend to be brief, which may not be conducive for explaining complex concepts. Although videos can be used to explain complex concepts, many Facebook users may not be able to view lengthy videos. Furthermore, some group members may not engage in the group (eg, “lurk”) or view all posts. Studies of Facebook interventions have shown that total group engagement tends to decline over time [65]. We leverage a number of strategies to enhance engagement, including use of polls, conversation starters, and Questions and Answers with experts in Facebook posts. Future studies may need to include only regular Facebook users. Second, recruiting a sufficiently large number of participants to populate multifamily groups of 50 to 60 family members within a relatively short period can be challenging. Timely enrollment is important when enrolling a cohort of 100 participants to randomize them into 2 groups. Finally, self-report sun protection measures show moderate correspondence with behavioral observations and diaries [92,93] and have moderate to high reproducibility and internal consistency [94-96].

### Strengths and Significance

Despite these limitations, this is the first randomized controlled trial to evaluate a Facebook family group intervention to improve surveillance and prevention for young melanoma survivors and their family members, an understudied at-risk population who are not adherent to risk-reduction recommendations. The use of SNA will offer insights into the normative influences in Facebook family groups. SNA, which is the study of the pattern of ties between social actors, allows for more than a simple count of the number of individuals in a person’s social network or the level of support that the individual receives from the network. SNA goes further to examine the social structures that emerge from the pattern of ties, and social network theory proposes that, through social influence processes, these network structures act as mechanisms that promote the adoption of health-related behaviors. We advance the science of social media–delivered interventions by examining the mechanisms by which the social networks that developed over the course of the Facebook intervention influence change. Although there is compelling evidence that behavior appears to “spread” through social networks, few studies (primarily in the laboratory setting) [97,98] have examined mechanisms by which this occurs. This knowledge will provide critical insights for improving the design of the next generation of web-based behavioral interventions. Finally, the use of key informant interviews with providers, cancer advocacy group leaders, and survivors and FDRs will elicit multiperspective, multilevel data regarding the acceptability, feasibility, appropriateness, and barriers to delivery and engagement in the Young Melanoma Family Facebook. From a “designing for dissemination” perspective [99], these qualitative findings will be essential in understanding the potential for and strategies needed to support widespread scale-up, dissemination, implementation, and maintenance of the intervention outside the research context, if it is found to be efficacious.

### Future Directions

The impact of this trial lies in its implementation and dissemination potential in a variety of contexts given the use of a familiar, popular, freely available social media platform. We will produce a content library of posts and a user guide that can guide implementation by others. If efficacious, the intervention could be disseminated and delivered via dermatologist practices, public health organizations such as the American Cancer Society, nonprofit organizations focused on melanoma, or in partnership with existing melanoma and skin cancer Facebook groups. The ability to interact with a skin cancer expert and other families affected by melanoma synchronously and asynchronously on Facebook may not only be convenient, but it may also be interesting to participants and may lead to a more impactful intervention. Knowledge gained will provide important information about potentially cost-effective ways to reach survivors and their families. If efficacious, future research should evaluate potential implementation strategies to reach young survivors and their family members with social media interventions.

## Appendix

Multimedia Appendix 1 CONSORT-eHEALTH checklist (V 1.6.1).

## Abbreviations

CSE: clinical skin examination
FDR: first-degree relative
IRB: Institutional Review Board
MLM: multilevel modeling
PI: principal investigator
SNA: social network analysis
SSE: skin self-examination

## Data Availability

The data sets generated during or analyzed during this study are available from the corresponding author on reasonable request.
