# Supplementary material for: Facebook Intervention for Young-Onset Melanoma Survivors and Families: Protocol for a Randomized Controlled Trial
Source: JMIR Res Protoc. 2023 Jan 24;12:e39640. doi: 10.2196/39640 (PMC9906316; doi:10.2196/39640)
Supplement: Multimedia Appendix 1 [file resprot_v12i1e39640_app1.pdf]

CONSORT-EHEALTH (V 1.6.1) - Submission/Publication Form

The CONSORT-EHEALTH checklist is intended for authors of randomized trials evaluating web-based and Internet-based applications/interventions, including mobile interventions, electronic games (incl multiplayer games), social media, certain telehealth applications, and other interactive and/or networked electronic applications. Some of the items (e.g. all subitems under item 5 - description of the intervention) may also be applicable for other study designs.

The goal of the CONSORT EHEALTH checklist and guideline is to be
a) a guide for reporting for authors of RCTs,
b) to form a basis for appraisal of an ehealth trial (in terms of validity)

CONSORT-EHEALTH items/subitems are MANDATORY reporting items for studies published in the Journal of Medical Internet Research and other journals / scientific societies endorsing the checklist.

Items numbered 1., 2., 3., 4a., 4b etc are original CONSORT or CONSORT-NPT (non-pharmacologic treatment) items.
Items with Roman numerals (i., ii, iii, iv etc.) are CONSORT-EHEALTH extensions/clarifications.

As the CONSORT-EHEALTH checklist is still considered in a formative stage, we would ask that you also RATE ON A SCALE OF 1-5 how important/useful you feel each item is FOR THE PURPOSE OF THE CHECKLIST and reporting guideline (optional).

Mandatory reporting items are marked with a red *.
In the textboxes, either copy & paste the relevant sections from your manuscript into this form - please include any quotes from your manuscript in QUOTATION MARKS,
or answer directly by providing additional information not in the manuscript, or elaborating on why the item was not relevant for this study.

YOUR ANSWERS WILL BE PUBLISHED AS A SUPPLEMENTARY FILE TO YOUR PUBLICATION IN JMIR AND ARE CONSIDERED PART OF YOUR PUBLICATION (IF ACCEPTED).
Please fill in these questions diligently. Information will not be copyedited, so please use proper spelling and grammar, use correct capitalization, and avoid abbreviations.

DO NOT FORGET TO SAVE AS PDF _AND_ CLICK THE SUBMIT BUTTON SO YOUR ANSWERS ARE IN OUR DATABASE !!!

Citation Suggestion (if you append the pdf as Appendix we suggest to cite this paper in the caption):
Eysenbach G, CONSORT-EHEALTH Group

CONSORT-EHEALTH: Improving and Standardizing Evaluation Reports of Web-based and Mobile Health Interventions
J Med Internet Res 2011;13(4):e126
URL: http://www.jmir.org/2011/4/e126/
doi: 10.2196/jmir.1923
PMID: 22209829

Sign in to Google to save your progress. Learn more

* Required

Your name *

First Last

Sharon Manne

Primary Affiliation (short), City, Country *

University of Toronto, Toronto, Canada

Rutgers Cancer Institute of New Jersey

Your e-mail address *

abc@gmail.com

Sharon.manne@rutgers.edu

Title of your manuscript *

Provide the (draft) title of your manuscript.

Facebook Intervention for Young Onset Melanoma Patients and Families: Protocol for a Randomized Controlled Trial

Name of your App/Software/Intervention *

If there is a short and a long/alternate name, write the short name first and add the long name in brackets.

Facebook

Evaluated Version (if any)

e.g. "V1", "Release 2017-03-01", "Version 2.0.27913"

385.0.0.32.114

Language(s) *

What language is the intervention/app in? If multiple languages are available, separate by comma (e.g. "English, French")

English

URL of your Intervention Website or App

e.g. a direct link to the mobile app on app in appstore (itunes, Google Play), or URL of the website. If the intervention is a DVD or hardware, you can also link to an Amazon page.

https://www.facebook.com/

URL of an image/screenshot (optional)

Your answer

Accessibility *

Can an enduser access the intervention presently?

- (x) access is free and open
- ( ) access only for special usergroups, not open
- ( ) access is open to everyone, but requires payment/subscription/in-app purchases
- ( ) app/intervention no longer accessible
- ( ) Other:

Primary Medical Indication/Disease/Condition *

e.g. "Stress", "Diabetes", or define the target group in brackets after the condition, e.g. "Autism (Parents of children with)", "Alzheimers (Informal Caregivers of)"

Melanoma

Primary Outcomes measured in trial *

comma-separated list of primary outcomes reported in the trial

This paper is a protocol, therefore our expecte

Secondary/other outcomes

Are there any other outcomes the intervention is expected to affect?

This paper is a protocol, therefore our expected secondary outcomes are: Skin Self Exams, and Sun protection behaviors, of young onset melanoma survivors

Recommended "Dose" *

What do the instructions for users say on how often the app should be used?

- ○ Approximately Daily
- ○ Approximately Weekly
- ○ Approximately Monthly
- ○ Approximately Yearly
- ○ "as needed"
- ◉ Other: Participants can access Facebook when they want.

Approx. Percentage of Users (starters) still using the app as recommended after 3 months *

- ◉ unknown / not evaluated
- ○ 0-10%
- ○ 11-20%
- ○ 21-30%
- ○ 31-40%
- ○ 41-50%
- ○ 51-60%
- ○ 61-70%
- ○ 71%-80%
- ○ 81-90%
- ○ 91-100%
- ○ Other:

Overall, was the app/intervention effective? *

- ◯ yes: all primary outcomes were significantly better in intervention group vs control
- ◯ partly: SOME primary outcomes were significantly better in intervention group vs control
- ◯ no statistically significant difference between control and intervention
- ◯ potentially harmful: control was significantly better than intervention in one or more outcomes
- ◯ inconclusive: more research is needed
- ◉ Other: This is a protocol paper we cannot assess effectiveness till after the t

Article Preparation Status/Stage *

At which stage in your article preparation are you currently (at the time you fill in this form)

- ◯ not submitted yet - in early draft status
- ◯ not submitted yet - in late draft status, just before submission
- ◯ submitted to a journal but not reviewed yet
- ◉ submitted to a journal and after receiving initial reviewer comments
- ◯ submitted to a journal and accepted, but not published yet
- ◯ published
- ◯ Other:

Journal *

If you already know where you will submit this paper (or if it is already submitted), please provide the journal name (if it is not JMIR, provide the journal name under "other")

- ○ not submitted yet / unclear where I will submit this
- ○ Journal of Medical Internet Research (JMIR)
- ○ JMIR mHealth and UHealth
- ○ JMIR Serious Games
- ○ JMIR Mental Health
- ○ JMIR Public Health
- ○ JMIR Formative Research
- ○ Other JMIR sister journal
- ◉ Other: JMIR Research Protocols (JRP).

Is this a full powered effectiveness trial or a pilot/feasibility trial? *

- ○ Pilot/feasibility
- ◉ Fully powered

Manuscript tracking number *

If this is a JMIR submission, please provide the manuscript tracking number under "other" (The ms tracking number can be found in the submission acknowledgement email, or when you login as author in JMIR. If the paper is already published in JMIR, then the ms tracking number is the four-digit number at the end of the DOI, to be found at the bottom of each published article in JMIR)

- ○ no ms number (yet) / not (yet) submitted to / published in JMIR
- ◉ Other: # 39640

TITLE AND ABSTRACT

1a) TITLE: Identification as a randomized trial in the title

1a) Does your paper address CONSORT item 1a? *

I.e does the title contain the phrase "Randomized Controlled Trial"? (if not, explain the reason under "other")

- (●) yes
- ( ) Other:

1a-i) Identify the mode of delivery in the title

Identify the mode of delivery. Preferably use “web-based” and/or “mobile” and/or “electronic game” in the title. Avoid ambiguous terms like “online”, “virtual”, “interactive”. Use “Internet-based” only if Intervention includes non-web-based Internet components (e.g. email), use “computer-based” or “electronic” only if offline products are used. Use “virtual” only in the context of “virtual reality” (3-D worlds). Use “online” only in the context of “online support groups”. Complement or substitute product names with broader terms for the class of products (such as “mobile” or “smart phone” instead of “iphone”), especially if the application runs on different platforms.

	1	2	3	4	5	
subitem not at all important	( )	( )	( )	( )	(●)	essential

Clear selection

Does your paper address subitem 1a-i? *

Copy and paste relevant sections from manuscript title (include quotes in quotation marks "like this" to indicate direct quotes from your manuscript), or elaborate on this item by providing additional information not in the ms, or briefly explain why the item is not applicable/relevant for your study

“Facebook Intervention for Young Onset Melanoma Patients and Families: Protocol for a Randomized Controlled Trial "

1a-ii) Non-web-based components or important co-interventions in title

Mention non-web-based components or important co-interventions in title, if any (e.g., “with telephone support”).

	1	2	3	4	5	
subitem not at all important	○	○	◉	○	○	essential

Clear selection

Does your paper address subitem 1a-ii?

Copy and paste relevant sections from manuscript title (include quotes in quotation marks "like this" to indicate direct quotes from your manuscript), or elaborate on this item by providing additional information not in the ms, or briefly explain why the item is not applicable/relevant for your study

N/A There were no non-web based components in the title

1a-iii) Primary condition or target group in the title

Mention primary condition or target group in the title, if any (e.g., “for children with Type I Diabetes”) Example: A Web-based and Mobile Intervention with Telephone Support for Children with Type I Diabetes: Randomized Controlled Trial

	1	2	3	4	5	
subitem not at all important	○	○	○	●	○	essential

Clear selection

Does your paper address subitem 1a-iii? *

Copy and paste relevant sections from manuscript title (include quotes in quotation marks "like this" to indicate direct quotes from your manuscript), or elaborate on this item by providing additional information not in the ms, or briefly explain why the item is not applicable/relevant for your study

“Facebook Intervention for Young Onset Melanoma Patients and Families: Protocol for a Randomized Controlled Trial "

1b) ABSTRACT: Structured summary of trial design, methods, results, and conclusions

NPT extension: Description of experimental treatment, comparator, care providers, centers, and blinding status.

1b-i) Key features/functionalities/components of the intervention and comparator in the METHODS section of the ABSTRACT

Mention key features/functionalities/components of the intervention and comparator in the abstract. If possible, also mention theories and principles used for designing the site. Keep in mind the needs of systematic reviewers and indexers by including important synonyms. (Note: Only report in the abstract what the main paper is reporting. If this information is missing from the main body of text, consider adding it)

	1	2	3	4	5	
subitem not at all important	○	○	○	○	◉	essential

Clear selection

Does your paper address subitem 1b-i? *

Copy and paste relevant sections from the manuscript abstract (include quotes in quotation marks "like this" to indicate direct quotes from your manuscript), or elaborate on this item by providing additional information not in the ms, or briefly explain why the item is not applicable/relevant for your study

“Methods: 583 survivors and 577 FDRs will be randomly assigned to either the Young Melanoma Family Facebook Group or the Melanoma Family Healthy Lifestyle Facebook Group condition. Participants complete measures of CSE, SSE, sun protection, and as mediator measures of relevant attitudes and beliefs pre- and post-intervention. The primary aim is to evaluate the impact of the Young Melanoma Family Facebook intervention versus the Melanoma Family Healthy Lifestyle Facebook intervention on CSE, SSE frequency and comprehensiveness, and sun protection among FDRs”

1b-ii) Level of human involvement in the METHODS section of the ABSTRACT

Clarify the level of human involvement in the abstract, e.g., use phrases like “fully automated” vs. “therapist/nurse/care provider/physician-assisted” (mention number and expertise of providers involved, if any). (Note: Only report in the abstract what the main paper is reporting. If this information is missing from the main body of text, consider adding it)

	1	2	3	4	5	
subitem not at all important	○	○	◉	○	○	essential

Clear selection

Does your paper address subitem 1b-ii?

Copy and paste relevant sections from the manuscript abstract (include quotes in quotation marks "like this" to indicate direct quotes from your manuscript), or elaborate on this item by providing additional information not in the ms, or briefly explain why the item is not applicable/relevant for your study

N/A Human involvement was not included

1b-iii) Open vs. closed, web-based (self-assessment) vs. face-to-face assessments in the METHODS section of the ABSTRACT

Mention how participants were recruited (online vs. offline), e.g., from an open access website or from a clinic or a closed online user group (closed usergroup trial), and clarify if this was a purely web-based trial, or there were face-to-face components (as part of the intervention or for assessment). Clearly say if outcomes were self-assessed through questionnaires (as common in web-based trials). Note: In traditional offline trials, an open trial (open-label trial) is a type of clinical trial in which both the researchers and participants know which treatment is being administered. To avoid confusion, use “blinded” or “unblinded” to indicated the level of blinding instead of “open”, as “open” in web-based trials usually refers to “open access” (i.e. participants can self-enrol). (Note: Only report in the abstract what the main paper is reporting. If this information is missing from the main body of text, consider adding it)

	1	2	3	4	5	
subitem not at all important	○	○	○	○	○	essential

Does your paper address subitem 1b-iii?

Copy and paste relevant sections from the manuscript abstract (include quotes in quotation marks "like this" to indicate direct quotes from your manuscript), or elaborate on this item by providing additional information not in the ms, or briefly explain why the item is not applicable/relevant for your study

All steps of the study are done via the web or phone services with no face-to-face assessment.

1b-iv) RESULTS section in abstract must contain use data

Report number of participants enrolled/assessed in each group, the use/uptake of the intervention (e.g., attrition/adherence metrics, use over time, number of logins etc.), in addition to primary/secondary outcomes. (Note: Only report in the abstract what the main paper is reporting. If this information is missing from the main body of text, consider adding it)

	1	2	3	4	5	
subitem not at all important	○	○	○	○	◉	essential

Clear selection

Does your paper address subitem 1b-iv?

Copy and paste relevant sections from the manuscript abstract (include quotes in quotation marks "like this" to indicate direct quotes from your manuscript), or elaborate on this item by providing additional information not in the ms, or briefly explain why the item is not applicable/relevant for your study

“Power calculations recommended a sample size of at least 577 survivors and 577 FDRs.”

1b-v) CONCLUSIONS/DISCUSSION in abstract for negative trials

Conclusions/Discussions in abstract for negative trials: Discuss the primary outcome - if the trial is negative (primary outcome not changed), and the intervention was not used, discuss whether negative results are attributable to lack of uptake and discuss reasons. (Note: Only report in the abstract what the main paper is reporting. If this information is missing from the main body of text, consider adding it)

	1	2	3	4	5	
subitem not at all important	○	○	○	◉	○	essential

Clear selection

Does your paper address subitem 1b-v?

Copy and paste relevant sections from the manuscript abstract (include quotes in quotation marks "like this" to indicate direct quotes from your manuscript), or elaborate on this item by providing additional information not in the ms, or briefly explain why the item is not applicable/relevant for your study

N/A , Project is still ongoing, therefore there are no results.

INTRODUCTION

2a) In INTRODUCTION: Scientific background and explanation of rationale

2a-i) Problem and the type of system/solution

Describe the problem and the type of system/solution that is object of the study: intended as stand-alone intervention vs. incorporated in broader health care program? Intended for a particular patient population? Goals of the intervention, e.g., being more cost-effective to other interventions, replace or complement other solutions? (Note: Details about the intervention are provided in “Methods” under 5)

	1	2	3	4	5	
subitem not at all important	○	○	◉	○	○	essential

Clear selection

Does your paper address subitem 2a-i? *

Copy and paste relevant sections from the manuscript (include quotes in quotation marks "like this" to indicate direct quotes from your manuscript), or elaborate on this item by providing additional information not in the ms, or briefly explain why the item is not applicable/relevant for your study

Problem:
“Despite the growing population of young melanoma survivors and their FDRs, engagement in CSE, SSE, and sun protection behaviors in this population remains low.”

“However, no studies have specifically targeted the growing population of young onset patients and their FDRs. Further, only one study has adopted a family-focused approach that delivers the intervention to both survivors and their FDRs.”

Solution:
“To address this gap, we developed the Young Melanoma Family Facebook intervention to increase performance of risk-reducing behaviors for young melanoma survivors and their FDRs. We selected a social media delivery platform for this intervention. Other than the online intervention evaluated by Bowen and colleagues [17], prior behavioral interventions for FDRs and patients have been delivered via individual print materials and/or telephone counseling [18, 22-26]. Our intervention work extends prior research by using a social media platform, Facebook, as the intervention delivery method. Given the ubiquitous use of social media, its potential for reach and communication presents a unique opportunity for health promotion interventions.”

2a-ii) Scientific background, rationale: What is known about the (type of) system

Scientific background, rationale: What is known about the (type of) system that is the object of the study (be sure to discuss the use of similar systems for other conditions/diagnoses, if appropiate), motivation for the study, i.e. what are the reasons for and what is the context for this specific study, from which stakeholder viewpoint is the study performed, potential impact of findings [2]. Briefly justify the choice of the comparator.

	1	2	3	4	5	
subitem not at all important	○	○	○	○	◉	essential

Clear selection

Does your paper address subitem 2a-ii? *

Copy and paste relevant sections from the manuscript (include quotes in quotation marks "like this" to indicate direct quotes from your manuscript), or elaborate on this item by providing additional information not in the ms, or briefly explain why the item is not applicable/relevant for your study

“Facebook was a particularly suitable way to deliver intervention content to a group of young melanoma survivors and their FDRs for two reasons. First, this demographic has high rates of Facebook use [33], and most Facebook users are already connected to family members via Facebook. Our multiple-family Facebook group intervention approach includes both young survivors and their family members, and thus addresses the unique needs of the young adult survivor population, who have higher needs for peer and family support [34-36], prefer to interact with other young adult survivors [33], and prefer to obtain support from peer survivors rather than from other sources [37]. Our use of Facebook as an intervention platform is also a good match for this populations’ preferences for information delivery, as they are high users of social media for health information [38].”

2b) In INTRODUCTION: Specific objectives or hypotheses

Does your paper address CONSORT subitem 2b? *

Copy and paste relevant sections from the manuscript (include quotes in quotation marks "like this" to indicate direct quotes from your manuscript), or elaborate on this item by providing additional information not in the ms, or briefly explain why the item is not applicable/relevant for your study

“The purpose of the current randomized clinical trial is to determine the efficacy of a Facebook intervention that provides information, goal setting, and peer support to increase CSE, SSE, and sun protection behaviors for young onset melanoma survivors and their FDRs.

Primary Aim. The primary aim is to examine the efficacy of the Young Melanoma Family Facebook intervention versus a Melanoma Family Healthy Lifestyle Facebook intervention on CSE (primary outcome), SSE frequency and comprehensiveness, sun protection behaviors, and indoor tanning (secondary outcomes) of FDRs of young onset melanoma survivors.

Hypothesis: Participants in the Young Melanoma Family Facebook intervention will report more CSE, more frequent and comprehensive SSEs, more sun protection behaviors and less tanning at the 6-month follow-up compared to those in the Melanoma Family Healthy Lifestyle Facebook condition.

Secondary Aim 1. One secondary aim is to examine the efficacy of the Young Melanoma Family Facebook intervention on survivors’ SSE frequency and comprehensiveness and sun protection behaviors. Survivors’ behaviors are considered a secondary aim because the vast majority are compliant with CSEs. However, many survivors are not adherent with comprehensive SSE and sun protection behaviors, and thus we will focus on these outcomes. Hypothesis: Survivor participants in the Young Melanoma Family Facebook intervention will report more frequent and comprehensive SSEs and higher sun protection at the 6-month follow-up compared to those in the Melanoma Family Healthy Lifestyle Facebook intervention condition.

Secondary Aim 2: Another secondary aim is to determine the mechanisms of intervention efficacy for both interventions’ impact on FDR and survivor outcomes.

Hypothesis: Based on the Theory of Normative Social Behavior [42] as well as the Preventive Health Model [39-41], we propose that the effects of the Young Melanoma Family Facebook intervention on FDR’s CSE, SSE, and sun protection behaviors and on survivors’ SSE and sun protection will be mediated by increased normative influences, family support and discussion, risk, benefits, barriers, self-efficacy, and use of change strategies.

Exploratory Aim: To evaluate the efficacy of the Melanoma Family Healthy Lifestyle Facebook intervention versus the Young Melanoma Family Facebook intervention on perceived stress, physical activity, and healthy eating patterns among FDRs and survivors.

Hypothesis: Participants in the Melanoma Family Healthy Lifestyle Facebook intervention will report less stress, more physical activity, and more healthy eating patterns at the 6-month follow-up compared to those in the Young Melanoma Family Facebook condition.”

METHODS

3a) Description of trial design (such as parallel, factorial) including allocation ratio

Does your paper address CONSORT subitem 3a? *

Copy and paste relevant sections from the manuscript (include quotes in quotation marks "like this" to indicate direct quotes from your manuscript), or elaborate on this item by providing additional information not in the ms, or briefly explain why the item is not applicable/relevant for your study

“This is a prospective, randomized controlled trial to evaluate the efficacy of the Young Melanoma Family Facebook intervention to increase engagement in CSE, SSE, and sun protection behaviors among young onset melanoma survivors and their first-degree relatives.”

“The randomization scheme was developed by the study statistician. Clusters of participants (survivor + FDRs) are randomly assigned to condition in blocks of 50 FDRs to achieve our target group size of approximately 25 survivors and 25 FDRs assigned to each group (Young Melanoma and Healthy Lifestyle). Members of the same family are assigned to the same condition. If additional FDRs join the study, they are assigned to the same condition. The team’s project coordinator allocates participants to their randomized condition and notifies them of study assignment via email.”

3b) Important changes to methods after trial commencement (such as eligibility criteria), with reasons

Does your paper address CONSORT subitem 3b? *

Copy and paste relevant sections from the manuscript (include quotes in quotation marks "like this" to indicate direct quotes from your manuscript), or elaborate on this item by providing additional information not in the ms, or briefly explain why the item is not applicable/relevant for your study

There have been no significant changes in protocol since the trial began.

3b-i) Bug fixes, Downtimes, Content Changes

Bug fixes, Downtimes, Content Changes: ehealth systems are often dynamic systems. A description of changes to methods therefore also includes important changes made on the intervention or comparator during the trial (e.g., major bug fixes or changes in the functionality or content) (5-iii) and other "unexpected events" that may have influenced study design such as staff changes, system failures/downtimes, etc. [2].

	1	2	3	4	5	
subitem not at all important	○	●	○	○	○	essential

Clear selection

Does your paper address subitem 3b-i?

Copy and paste relevant sections from the manuscript (include quotes in quotation marks "like this" to indicate direct quotes from your manuscript), or elaborate on this item by providing additional information not in the ms, or briefly explain why the item is not applicable/relevant for your study

N/A, The trial conditions are administered online via groups on Facebook. Facebook's website very rarely has significant outages, or major changes to its functionality outside of minor user interface changes. Therefore, it is unlikely that any of these factors could have affected outcomes.

4a) Eligibility criteria for participants

Does your paper address CONSORT subitem 4a? *

Copy and paste relevant sections from the manuscript (include quotes in quotation marks "like this" to indicate direct quotes from your manuscript), or elaborate on this item by providing additional information not in the ms, or briefly explain why the item is not applicable/relevant for your study

The following eligibility criteria was taken from table 1:
Survivor eligibility:
Inclusion Criteria:

- Diagnosed with stage 0-3 melanoma in the last 5 years
- Age at diagnosis 18-39 years
- Completed treatment at least 3 months previously
- At least one family member consents

Exclusion Criteria:

- Concurrent cancer diagnosis
- Cannot speak and read English
- Does not have access to computer, internet, and has a Facebook account

Physical Activity Readiness Questionnaire [94]: This is a 7-item scale that used as a disclaimer to identify individuals who should consult a physician for medical clearance (if they mark yes to any of the 7 items) before engaging in physical activity in the event they are in the control group.

FDR Eligibility:
Inclusion:

- Current age 18-80 years
- First degree relative of survivor
- Survivor consents to the study
- Has only one first degree relative with melanoma (survivor)

Has not had a clinical skin exam in the past 3 years, has done SSE fewer than three times in the past year, OR has a sun protection habits average score less than or equal to 4 (“often”)
Exclusion:

- Does not have a personal history of melanoma
- Cannot speak and read English
- Does not have access to computer, internet, and has a Facebook account

Physical Activity Readiness Questionnaire [94]: This is a 7-item scale that used as a disclaimer to identify individuals who should consult a physician for medical clearance before engaging in physical activity in the event they are in the control group.

4a-i) Computer / Internet literacy

Computer / Internet literacy is often an implicit "de facto" eligibility criterion - this should be explicitly clarified.

	1	2	3	4	5	
subitem not at all important	○	◉	○	○	○	essential

Clear selection

Does your paper address subitem 4a-i?

Copy and paste relevant sections from the manuscript (include quotes in quotation marks "like this" to indicate direct quotes from your manuscript), or elaborate on this item by providing additional information not in the ms, or briefly explain why the item is not applicable/relevant for your study

This is implied given they have to be on Facebook and complete an online survey and consent form.

4a-ii) Open vs. closed, web-based vs. face-to-face assessments:

Open vs. closed, web-based vs. face-to-face assessments: Mention how participants were recruited (online vs. offline), e.g., from an open access website or from a clinic, and clarify if this was a purely web-based trial, or there were face-to-face components (as part of the intervention or for assessment), i.e., to what degree got the study team to know the participant. In online-only trials, clarify if participants were quasi-anonymous and whether having multiple identities was possible or whether technical or logistical measures (e.g., cookies, email confirmation, phone calls) were used to detect/prevent these.

	1	2	3	4	5	
subitem not at all important	○	○	○	○	◉	essential

Clear selection

Does your paper address subitem 4a-ii? *

Copy and paste relevant sections from the manuscript (include quotes in quotation marks "like this" to indicate direct quotes from your manuscript), or elaborate on this item by providing additional information not in the ms, or briefly explain why the item is not applicable/relevant for your study

“Survivors who are eligible are sent an electronic link to the informed consent. Once eligible, survivors supply the names, emails, and phone numbers of their parents, children over 18, and full siblings. FDRs are contacted by telephone and eligibility is confirmed. If eligible, FDRs are invited to participate. If interested, the electronic link to the consent and survey is provided by email. For both survivors and FDRs, calls are made on four evenings per week and on weekends to ensure participants are reached. “

Participants were recruited and underwent the study via the internet and phone. No face-to-face assessments occurred. Participants were required to have a valid Facebook account to view study intervention materials, as well as provide information on themselves and their FDR, therefore it is very unlikely that individuals were able to complete the study multiple times under fake accounts.

4a-iii) Information giving during recruitment

Information given during recruitment. Specify how participants were briefed for recruitment and in the informed consent procedures (e.g., publish the informed consent documentation as appendix, see also item X26), as this information may have an effect on user self-selection, user expectation and may also bias results.

	1	2	3	4	5	
subitem not at all important	○	○	○	◉	○	essential

Clear selection

Does your paper address subitem 4a-iii?

Copy and paste relevant sections from the manuscript (include quotes in quotation marks "like this" to indicate direct quotes from your manuscript), or elaborate on this item by providing additional information not in the ms, or briefly explain why the item is not applicable/relevant for your study

“To confirm eligibility prior to enrollment, staff administer a series of screening questions to interested participants. During eligibility screening, all survivors are asked to verify their date of birth, date of cancer diagnosis, internet access, and Facebook account status. All FDRs are asked to confirm their date of birth, internet access, and Facebook account status.”

“First, the study’s informed consent documentation process includes a description of the privacy settings for Facebook groups. Second, during a phone contact with each participant prior to the group’s initiation, the research staff describes what a “secret” Facebook group means in terms of privacy (e.g., group and its contents are not visible to the public), the privacy limitations for a “secret” Facebook group (e.g., cannot guarantee other participants won’t share content posted with others), and the confidentiality rules of the group.”

4b) Settings and locations where the data were collected

Does your paper address CONSORT subitem 4b? *

Copy and paste relevant sections from the manuscript (include quotes in quotation marks "like this" to indicate direct quotes from your manuscript), or elaborate on this item by providing additional information not in the ms, or briefly explain why the item is not applicable/relevant for your study

N/A. Data was collected from participants across the country through online tools. Therefore, there is no specific location or setting for data collection.

4b-i) Report if outcomes were (self-)assessed through online questionnaires

Clearly report if outcomes were (self-)assessed through online questionnaires (as common in web-based trials) or otherwise.

	1	2	3	4	5	
subitem not at all important	○	○	○	○	●	essential

Clear selection

Does your paper address subitem 4b-i? *

Copy and paste relevant sections from the manuscript (include quotes in quotation marks "like this" to indicate direct quotes from your manuscript), or elaborate on this item by providing additional information not in the ms, or briefly explain why the item is not applicable/relevant for your study

“After group completion, participants are sent a link for the first follow-up survey and intervention evaluation, which is completed online. Three months after group completion, participants are sent a link for the second follow-up survey, which is completed online.”

4b-ii) Report how institutional affiliations are displayed

Report how institutional affiliations are displayed to potential participants [on ehealth media], as affiliations with prestigious hospitals or universities may affect volunteer rates, use, and reactions with regards to an intervention.(Not a required item – describe only if this may bias results)

	1	2	3	4	5	
subitem not at all important	●	○	○	○	○	essential

Clear selection

Does your paper address subitem 4b-ii?

Copy and paste relevant sections from the manuscript (include quotes in quotation marks "like this" to indicate direct quotes from your manuscript), or elaborate on this item by providing additional information not in the ms, or briefly explain why the item is not applicable/relevant for your study

N/A we do not believe that the affiliation would create bias.

5) The interventions for each group with sufficient details to allow replication, including how and when they were actually administered

5-i) Mention names, credential, affiliations of the developers, sponsors, and owners

Mention names, credential, affiliations of the developers, sponsors, and owners [6] (if authors/evaluators are owners or developer of the software, this needs to be declared in a "Conflict of interest" section or mentioned elsewhere in the manuscript).

	1	2	3	4	5	
subitem not at all important	○	●	○	○	○	essential

Clear selection

Does your paper address subitem 5-i?

Copy and paste relevant sections from the manuscript (include quotes in quotation marks "like this" to indicate direct quotes from your manuscript), or elaborate on this item by providing additional information not in the ms, or briefly explain why the item is not applicable/relevant for your study

The software being use to deliver the intervention, Facebook, was not developed by any study team members or relatives. In addition all authors, funding sources, and affiliations, as well as a conflict of interest statement can be found on the first two pages of the submitted document.

5-ii) Describe the history/development process

Describe the history/development process of the application and previous formative evaluations (e.g., focus groups, usability testing), as these will have an impact on adoption/use rates and help with interpreting results.

	1	2	3	4	5	
subitem not at all important	○	○	○	◉	○	essential

Clear selection

Does your paper address subitem 5-ii?

Copy and paste relevant sections from the manuscript (include quotes in quotation marks "like this" to indicate direct quotes from your manuscript), or elaborate on this item by providing additional information not in the ms, or briefly explain why the item is not applicable/relevant for your study

As the study is still ongoing there has not yet been significant development changes, however there are plans to implement stakeholder interviews, as pasted below:

“To inform future potential dissemination and implementation of the Young Melanoma Family Facebook Group intervention, we will conduct semi-structured key informant interviews with three types of stakeholders: (1) 5 providers (cutaneous oncologists, dermatologists); (2) 5 cancer advocacy group leaders (e.g., Stupid Cancer); and (3) 30 study participants (survivors and FDRs). Study participants will be purposively selected from the Young Melanoma Family Facebook Group condition, including approximately 15 survivors and 15 FDRs, and representing variation in participation in the intervention and scores on the 18 intervention evaluation items. Interviews (45-60 minutes in length) will be conducted using videoconferencing software and audio-recorded with participant permission. Interview participants will be compensated $50.”

5-iii) Revisions and updating

Revisions and updating. Clearly mention the date and/or version number of the application/intervention (and comparator, if applicable) evaluated, or describe whether the intervention underwent major changes during the evaluation process, or whether the development and/or content was “frozen” during the trial. Describe dynamic components such as news feeds or changing content which may have an impact on the replicability of the intervention (for unexpected events see item 3b).

	1	2	3	4	5	
subitem not at all important	○	○	◉	○	○	essential

Clear selection

Does your paper address subitem 5-iii?

Copy and paste relevant sections from the manuscript (include quotes in quotation marks "like this" to indicate direct quotes from your manuscript), or elaborate on this item by providing additional information not in the ms, or briefly explain why the item is not applicable/relevant for your study

N/A. The intervention is administered via Facebook, which very rarely changes its functionality aside from occasional user interface changes.

5-iv) Quality assurance methods

Provide information on quality assurance methods to ensure accuracy and quality of information provided [1], if applicable.

	1	2	3	4	5	
subitem not at all important	○	○	○	◉	○	essential

Clear selection

Does your paper address subitem 5-iv?

Copy and paste relevant sections from the manuscript (include quotes in quotation marks "like this" to indicate direct quotes from your manuscript), or elaborate on this item by providing additional information not in the ms, or briefly explain why the item is not applicable/relevant for your study

“We will use an intent-to-treat approach to analyses of primary and secondary outcomes. Our approach to recruitment and retention minimizes missing data by contacting participants who do not complete sections of the survey, and by incentivizing participation. Initial analyses will examine characteristics of non-completers. As recommended by Lang & Little [76], multiple imputation (using 50 imputed samples) will be used to impute missing values.”

5-v) Ensure replicability by publishing the source code, and/or providing screenshots/screen-capture video, and/or providing flowcharts of the algorithms used

Ensure replicability by publishing the source code, and/or providing screenshots/screen-capture video, and/or providing flowcharts of the algorithms used. Replicability (i.e., other researchers should in principle be able to replicate the study) is a hallmark of scientific reporting.

	1	2	3	4	5	
subitem not at all important	○	◉	○	○	○	essential

Clear selection

Does your paper address subitem 5-v?

Copy and paste relevant sections from the manuscript (include quotes in quotation marks "like this" to indicate direct quotes from your manuscript), or elaborate on this item by providing additional information not in the ms, or briefly explain why the item is not applicable/relevant for your study

This intervention is done on Facebook, which is a free, easily accessible, and commonly used website. In addition, examples of posts are presented in the attached document.

5-vi) Digital preservation

Digital preservation: Provide the URL of the application, but as the intervention is likely to change or disappear over the course of the years; also make sure the intervention is archived (Internet Archive, webcitation.org, and/or publishing the source code or screenshots/videos alongside the article). As pages behind login screens cannot be archived, consider creating demo pages which are accessible without login.

	1	2	3	4	5	
subitem not at all important	(●)	( )	( )	( )	( )	essential

Clear selection

Does your paper address subitem 5-vi?

Copy and paste relevant sections from the manuscript (include quotes in quotation marks "like this" to indicate direct quotes from your manuscript), or elaborate on this item by providing additional information not in the ms, or briefly explain why the item is not applicable/relevant for your study

Since Participants join these Facebook groups using their personal accounts, which can include their real names and other personal information, access to the group cannot be archived or shared as it would be a breach of confidentiality.

5-vii) Access

Access: Describe how participants accessed the application, in what setting/context, if they had to pay (or were paid) or not, whether they had to be a member of specific group. If known, describe how participants obtained “access to the platform and Internet” [1]. To ensure access for editors/reviewers/readers, consider to provide a “backdoor” login account or demo mode for reviewers/readers to explore the application (also important for archiving purposes, see vi).

	1	2	3	4	5	
subitem not at all important	( )	( )	( )	(●)	( )	essential

Clear selection

Does your paper address subitem 5-vii? *

Copy and paste relevant sections from the manuscript (include quotes in quotation marks "like this" to indicate direct quotes from your manuscript), or elaborate on this item by providing additional information not in the ms, or briefly explain why the item is not applicable/relevant for your study

“Upon family randomization, participants are provided with the Facebook name of the intervention account they have been randomly assigned to and asked to “friend” that respective account. Once participants “friend” the Facebook group account, staff post a link into the intervention Facebook group account? that participants use to authorize the Grytics software to capture their engagement data [46]. When a participant clicks the opt-in button, it redirects the participant to their own Facebook settings and provides more details about the information the app will be collecting (Facebook name, profile picture, posts, comments, reactions). The participants have the option to indicate which of their Facebook groups the Grytics software can see by clicking “choose what you allow.” When the participant accepts the invite, their request to join the group stays in “pending” status until the group administrator approves all members on Day 1 of the group. Those who do not “friend” the Facebook accounts are contacted by study staff.”

5-viii) Mode of delivery, features/functionalities/components of the intervention and comparator, and the theoretical framework

Describe mode of delivery, features/functionalities/components of the intervention and comparator, and the theoretical framework [6] used to design them (instructional strategy [1], behaviour change techniques, persuasive features, etc., see e.g., [7, 8] for terminology). This includes an in-depth description of the content (including where it is coming from and who developed it) [1],” whether [and how] it is tailored to individual circumstances and allows users to track their progress and receive feedback” [6]. This also includes a description of communication delivery channels and – if computer-mediated communication is a component – whether communication was synchronous or asynchronous [6]. It also includes information on presentation strategies [1], including page design principles, average amount of text on pages, presence of hyperlinks to other resources, etc. [1].

	1	2	3	4	5	
subitem not at all important	○	○	◉	○	○	essential

Clear selection

Does your paper address subitem 5-viii? *

Copy and paste relevant sections from the manuscript (include quotes in quotation marks "like this" to indicate direct quotes from your manuscript), or elaborate on this item by providing additional information not in the ms, or briefly explain why the item is not applicable/relevant for your study

"Young Melanoma Family Facebook Group
Participants are asked to join a secret Facebook group where membership and content is only viewable by invited group members. Participants' group membership and activities are not publicly viewable to outside Facebook users. Groups run for 12 weeks and contain up to 54 members (25 survivors and their FDRs). Posts are made twice a day for the first 8 weeks of the group, which is the frequency suggested by social media marketers to engage participants without overburdening them [47]. For weeks 9 to 12, four to six posts are made each week. Each week is organized around one of the following topics: melanoma risk, physician skin exam, skin self-check, and sun protection habits . The first four weeks are organized according to the following topics: risk factors, CSE, skin self-check, and sun protection habits. Behavior change strategies such as goal setting and increasing support for CSE/SSE/sun protection are woven into posts. During Weeks 5-8, content is expanded for each topic, special topics are addressed, and news and stories about skin cancer are presented. Messages reinforce regular sun protection, clinician skin exam, skin self-exams (e.g., mole map pictures), and group discussion. The surgical oncologist and content expert on skin cancer (A. Berger) replies to the "Ask the Expert" postings in the Young Melanoma Family Group, for questions that are not already included in the Question and Answer Library. Engagement is enhanced by engaging and humorous pictures, links to educational materials, polls, questions ("What is the ultraviolet light index in your town today?" "What is your favorite brand of sunscreen?"), memes, and holiday greetings. Each group ends at 12 weeks at which time participants are no longer able to post. However, participants will be able to review the previous 12 weeks of postings"

"Melanoma Family Healthy Lifestyle Facebook Group
Procedures for joining the group, Facebook privacy settings, moderation guidelines, frequency of posts, group duration, and access after the group ends are the same as for the Young Melanoma Family Facebook group. Each week is organized around one of the following topics: nutrition, physical activity, sleep, stress . Sample posts are shown in Figure 3. Engagement is enhanced by including humorous memes, polls, conversation starters, and seasonal greetings."

5-ix) Describe use parameters

Describe use parameters (e.g., intended “doses” and optimal timing for use). Clarify what instructions or recommendations were given to the user, e.g., regarding timing, frequency, heaviness of use, if any, or was the intervention used ad libitum.

	1	2	3	4	5	
subitem not at all important	○	○	◉	○	○	essential

Clear selection

Does your paper address subitem 5-ix?

Copy and paste relevant sections from the manuscript (include quotes in quotation marks "like this" to indicate direct quotes from your manuscript), or elaborate on this item by providing additional information not in the ms, or briefly explain why the item is not applicable/relevant for your study

“Posts are made twice a day for the first 8 weeks of the group, which is the frequency suggested by social media marketers to engage participants without overburdening them [47]. For weeks 9 to 12, four to six posts are made each week”

5-x) Clarify the level of human involvement

Clarify the level of human involvement (care providers or health professionals, also technical assistance) in the e-intervention or as co-intervention (detail number and expertise of professionals involved, if any, as well as “type of assistance offered, the timing and frequency of the support, how it is initiated, and the medium by which the assistance is delivered”. It may be necessary to distinguish between the level of human involvement required for the trial, and the level of human involvement required for a routine application outside of a RCT setting (discuss under item 21 – generalizability).

	1	2	3	4	5	
subitem not at all important	○	○	○	○	◉	essential

Clear selection

Does your paper address subitem 5-x?

Copy and paste relevant sections from the manuscript (include quotes in quotation marks "like this" to indicate direct quotes from your manuscript), or elaborate on this item by providing additional information not in the ms, or briefly explain why the item is not applicable/relevant for your study

“The moderator logs in two to three times a day to review activity and facilitate engagement by “liking” participant comments and replying with supportive responses. Because Facebook norms are for brief and frequent interactions, moderator responses will be brief, but engage participants in discussions, reinforce engagement (e.g., using a “like” reaction), and answer questions. Study collaborator Pagoto reviews Facebook activity on a regular basis.”

“In terms of appropriate posts by members, the moderator notifies the Principal Investigator (Manne) if unanticipated, distressing, or inappropriate comments are posted in the Facebook group. The study team will discuss the appropriate response to the issue and will provide guidance to the moderator on how best to proceed.”

5-xi) Report any prompts/reminders used

Report any prompts/reminders used: Clarify if there were prompts (letters, emails, phone calls, SMS) to use the application, what triggered them, frequency etc. It may be necessary to distinguish between the level of prompts/reminders required for the trial, and the level of prompts/reminders for a routine application outside of a RCT setting (discuss under item 21 – generalizability).

	1	2	3	4	5	
subitem not at all important	○	○	○	●	○	essential

Clear selection

Does your paper address subitem 5-xi? *

Copy and paste relevant sections from the manuscript (include quotes in quotation marks "like this" to indicate direct quotes from your manuscript), or elaborate on this item by providing additional information not in the ms, or briefly explain why the item is not applicable/relevant for your study

“For both survivors and FDRs, calls are made on four evenings per week and on weekends to ensure participants are reached. Up to ten attempts are made to contact prospective participants by phone before they are deemed unable to be located.”

“The data management program, DatStat, is programmed to send three reminder emails. Staff complete a minimum of one phone call for incomplete surveys.”

5-xii) Describe any co-interventions (incl. training/support)

Describe any co-interventions (incl. training/support): Clearly state any interventions that are provided in addition to the targeted eHealth intervention, as ehealth intervention may not be designed as stand-alone intervention. This includes training sessions and support [1]. It may be necessary to distinguish between the level of training required for the trial, and the level of training for a routine application outside of a RCT setting (discuss under item 21 – generalizability.

	1	2	3	4	5	
subitem not at all important	○	○	○	○	◉	essential

Clear selection

Does your paper address subitem 5-xii? *

Copy and paste relevant sections from the manuscript (include quotes in quotation marks "like this" to indicate direct quotes from your manuscript), or elaborate on this item by providing additional information not in the ms, or briefly explain why the item is not applicable/relevant for your study

N/A no other interventions were being performed.

6a) Completely defined pre-specified primary and secondary outcome measures, including how and when they were assessed

Does your paper address CONSORT subitem 6a? *

Copy and paste relevant sections from the manuscript (include quotes in quotation marks "like this" to indicate direct quotes from your manuscript), or elaborate on this item by providing additional information not in the ms, or briefly explain why the item is not applicable/relevant for your study

Yes, all primary and secondary outcome measures were listed and explained within the methods section. Due to the large number of measures they were not copy and pasted into this text box, however they can be found on pages 16 though 25 in the submitted document.

6a-i) Online questionnaires: describe if they were validated for online use and apply CHERRIES items to describe how the questionnaires were designed/deployed

If outcomes were obtained through online questionnaires, describe if they were validated for online use and apply CHERRIES items to describe how the questionnaires were designed/deployed [9].

	1	2	3	4	5	
subitem not at all important	○	◉	○	○	○	essential

Clear selection

Does your paper address subitem 6a-i?

Copy and paste relevant sections from manuscript text

N/A

6a-ii) Describe whether and how “use” (including intensity of use/dosage) was defined/measured/monitored

Describe whether and how “use” (including intensity of use/dosage) was defined/measured/monitored (logins, logfile analysis, etc.). Use/adoption metrics are important process outcomes that should be reported in any ehealth trial.

	1	2	3	4	5	
subitem not at all important	○	○	◉	○	○	essential

Clear selection

Does your paper address subitem 6a-ii?

Copy and paste relevant sections from manuscript text

“General use of and connectedness with Facebook. Facebook usage will be measured with three items (e.g., log-ins per week, membership in private or public groups). Participants will complete the Facebook Intensity scale [73], which consists of a five-item experiential measure of Facebook usage separate from behavioral measures of frequency and duration, incorporating emotional connectedness to the site and its integration into individuals’ daily activities (e.g., “I feel I am part of the Facebook community.”) Ratings range from 1=strongly disagree, 5=strongly agree, and higher scores indicate more connection with Facebook.

Facebook group engagement. Facebook data will be extracted to assess behavioral engagement using Grytics extraction software to capture views, likes, and comments to posts. We will calculate descriptive information on each post, each group cohort, and by individual participants. Participant engagement (i.e., reactions, replies, poll votes, and posts) will be analyzed as a predictor of response to treatment in the analyses.”

6a-iii) Describe whether, how, and when qualitative feedback from participants was obtained

Describe whether, how, and when qualitative feedback from participants was obtained (e.g., through emails, feedback forms, interviews, focus groups).

	1	2	3	4	5	
subitem not at all important	○	○	○	●	○	essential

Clear selection

Does your paper address subitem 6a-iii?

Copy and paste relevant sections from manuscript text

“To inform future potential dissemination and implementation of the Young Melanoma Family Facebook Group intervention, we will conduct semi-structured key informant interviews with three types of stakeholders: (1) 5 providers (cutaneous oncologists, dermatologists); (2) 5 cancer advocacy group leaders (e.g., Stupid Cancer); and (3) 30 study participants (survivors and FDRs). Study participants will be purposively selected from the Young Melanoma Family Facebook Group condition, including approximately 15 survivors and 15 FDRs, and representing variation in participation in the intervention and scores on the 18 intervention evaluation items. Interviews (45-60 minutes in length) will be conducted using videoconferencing software and audio-recorded with participant permission. Interview participants will be compensated $50.

Interview guides will be tailored to each of the three stakeholder types to assess multiple perspectives on the acceptability, feasibility, and appropriateness of the intervention. Providers and cancer advocacy group leaders will be introduced to the intervention at the beginning of their interviews. Interview questions and probes (as appropriate for each stakeholder group) will focus on: (1) current approaches and practices to promote CSE, SSE, and sun protection; (2) perceived benefits and shortcomings of the Young Melanoma Family Facebook intervention; (3) actual and potential barriers to its use and delivery; (4) possible dissemination channels[74] to reach survivors and their families (e.g., communication modes, provider recommendation, social marketing); (5) pros and cons of suggested dissemination channels and strategies; (6) the “fit” of the intervention with the preferences of survivors and FDRs and the missions and objectives of providers and organizations [75]; and (7) resources, infrastructure, and other factors needed to implement and sustain the intervention outside of the research context.”

6b) Any changes to trial outcomes after the trial commenced, with reasons

Does your paper address CONSORT subitem 6b? *

Copy and paste relevant sections from the manuscript (include quotes in quotation marks "like this" to indicate direct quotes from your manuscript), or elaborate on this item by providing additional information not in the ms, or briefly explain why the item is not applicable/relevant for your study

N/A no changes to trial outcomes since it commenced.

7a) How sample size was determined

NPT: When applicable, details of whether and how the clustering by care provides or centers was addressed

7a-i) Describe whether and how expected attrition was taken into account when calculating the sample size

Describe whether and how expected attrition was taken into account when calculating the sample size.

	1	2	3	4	5	
subitem not at all important	○	○	○	◉	○	essential

Clear selection

Does your paper address subitem 7a-i?

Copy and paste relevant sections from manuscript title (include quotes in quotation marks "like this" to indicate direct quotes from your manuscript), or elaborate on this item by providing additional information not in the ms, or briefly explain why the item is not applicable/relevant for your study

“Sample size calculations for mediated effects are based on simulation studies that model the two pieces of the mediating path (study arm to mediator; mediator to outcome) [81]. Assuming 583 survivors with 22% dropout, there is greater than 80% power to detect paths with regression coefficients of .33 (small-to-moderate effects) [82]. Assuming 577 FDRs with 22% dropout, there is 80% power to detect paths with regression coefficients between 0.20-0.23 (small effect sizes). With smaller ICCs, effect sizes may be closer to 0.20.”

7b) When applicable, explanation of any interim analyses and stopping guidelines

Does your paper address CONSORT subitem 7b? *

Copy and paste relevant sections from the manuscript (include quotes in quotation marks "like this" to indicate direct quotes from your manuscript), or elaborate on this item by providing additional information not in the ms, or briefly explain why the item is not applicable/relevant for your study

N/A study is still under progress, and there has been no interim analyses

8a) Method used to generate the random allocation sequence

NPT: When applicable, how care providers were allocated to each trial group

Does your paper address CONSORT subitem 8a? *

Copy and paste relevant sections from the manuscript (include quotes in quotation marks "like this" to indicate direct quotes from your manuscript), or elaborate on this item by providing additional information not in the ms, or briefly explain why the item is not applicable/relevant for your study

“The randomization scheme was developed by the study statistician. Clusters of participants (survivor + FDRs) are randomly assigned to condition in blocks of 50 FDRs to achieve our target group size of approximately 25 survivors and 25 FDRs assigned to each group (Young Melanoma and Healthy Lifestyle). Members of the same family are assigned to the same condition. If additional FDRs join the study, they are assigned to the same condition. The team’s project coordinator allocates participants to their randomized condition and notifies them of study assignment via email.”

8b) Type of randomisation; details of any restriction (such as blocking and block size)

Does your paper address CONSORT subitem 8b? *

Copy and paste relevant sections from the manuscript (include quotes in quotation marks "like this" to indicate direct quotes from your manuscript), or elaborate on this item by providing additional information not in the ms, or briefly explain why the item is not applicable/relevant for your study

“The randomization scheme was developed by the study statistician. Clusters of participants (survivor + FDRs) are randomly assigned to condition in blocks of 50 FDRs to achieve our target group size of approximately 25 survivors and 25 FDRs assigned to each group (Young Melanoma and Healthy Lifestyle). Members of the same family are assigned to the same condition. If additional FDRs join the study, they are assigned to the same condition. The team’s project coordinator allocates participants to their randomized condition and notifies them of study assignment via email.”

9) Mechanism used to implement the random allocation sequence (such as sequentially numbered containers), describing any steps taken to conceal the sequence until interventions were assigned

Does your paper address CONSORT subitem 9? *

Copy and paste relevant sections from the manuscript (include quotes in quotation marks "like this" to indicate direct quotes from your manuscript), or elaborate on this item by providing additional information not in the ms, or briefly explain why the item is not applicable/relevant for your study

“The randomization scheme was developed by the study statistician. Clusters of participants (survivor + FDRs) are randomly assigned to condition in blocks of 50 FDRs to achieve our target group size of approximately 25 survivors and 25 FDRs assigned to each group (Young Melanoma and Healthy Lifestyle). Members of the same family are assigned to the same condition. If additional FDRs join the study, they are assigned to the same condition. The team’s project coordinator allocates participants to their randomized condition and notifies them of study assignment via email.”

10) Who generated the random allocation sequence, who enrolled participants, and who assigned participants to interventions

Does your paper address CONSORT subitem 10? *

Copy and paste relevant sections from the manuscript (include quotes in quotation marks "like this" to indicate direct quotes from your manuscript), or elaborate on this item by providing additional information not in the ms, or briefly explain why the item is not applicable/relevant for your study

“The randomization scheme was developed by the study statistician. Clusters of participants (survivor + FDRs) are randomly assigned to condition in blocks of 50 FDRs to achieve our target group size of approximately 25 survivors and 25 FDRs assigned to each group (Young Melanoma and Healthy Lifestyle). Members of the same family are assigned to the same condition. If additional FDRs join the study, they are assigned to the same condition. The team’s project coordinator allocates participants to their randomized condition and notifies them of study assignment via email.”

11a) If done, who was blinded after assignment to interventions (for example, participants, care providers, those assessing outcomes) and how

NPT: Whether or not administering co-interventions were blinded to group assignment

11a-i) Specify who was blinded, and who wasn't

Specify who was blinded, and who wasn't. Usually, in web-based trials it is not possible to blind the participants [1, 3] (this should be clearly acknowledged), but it may be possible to blind outcome assessors, those doing data analysis or those administering co-interventions (if any).

	1	2	3	4	5	
subitem not at all important	○	○	○	●	○	essential

Clear selection

Does your paper address subitem 11a-i? *

Copy and paste relevant sections from the manuscript (include quotes in quotation marks "like this" to indicate direct quotes from your manuscript), or elaborate on this item by providing additional information not in the ms, or briefly explain why the item is not applicable/relevant for your study

Study participants were not blinded

11a-ii) Discuss e.g., whether participants knew which intervention was the "intervention of interest" and which one was the "comparator"

Informed consent procedures (4a-ii) can create biases and certain expectations - discuss e.g., whether participants knew which intervention was the "intervention of interest" and which one was the "comparator".

	1	2	3	4	5	
subitem not at all important	○	○	●	○	○	essential

Clear selection

Does your paper address subitem 11a-ii?

Copy and paste relevant sections from the manuscript (include quotes in quotation marks "like this" to indicate direct quotes from your manuscript), or elaborate on this item by providing additional information not in the ms, or briefly explain why the item is not applicable/relevant for your study

Participants do not know which arm is the comparator.

11b) If relevant, description of the similarity of interventions

(this item is usually not relevant for ehealth trials as it refers to similarity of a placebo or sham intervention to a active medication/intervention)

Does your paper address CONSORT subitem 11b? *

Copy and paste relevant sections from the manuscript (include quotes in quotation marks "like this" to indicate direct quotes from your manuscript), or elaborate on this item by providing additional information not in the ms, or briefly explain why the item is not applicable/relevant for your study

As mentioned below, both interventions followed a similar method of delivery and use of Facebook, the only difference being the content posted by team members.

“Procedures for joining the group, Facebook privacy settings, moderation guidelines, frequency of posts, group duration, and access after the group ends are the same as for the Young Melanoma Family Facebook group”

12a) Statistical methods used to compare groups for primary and secondary outcomes

NPT: When applicable, details of whether and how the clustering by care providers or centers was addressed

Does your paper address CONSORT subitem 12a? *

Copy and paste relevant sections from the manuscript (include quotes in quotation marks "like this" to indicate direct quotes from your manuscript), or elaborate on this item by providing additional information not in the ms, or briefly explain why the item is not applicable/relevant for your study

“These two aims (Primary Aim, Secondary Aim 1) will be addressed together using multilevel modeling (MLM) because the data from FDRs and survivors are assumed to be interdependent. The exploratory aim of testing whether the intervention is effective in reducing perceived stress and improving other outcomes such as physical activity will also use this approach. MLMs with the following attributes will be used to address the primary aim and secondary aim 1:

•Family is the upper-level sampling unit and individual (FDR or survivor) is the lower-level unit

•Fixed effects variables include condition (Young Melanoma versus Melanoma Family Healthy Lifestyle Facebook), respondent role (parent, sibling, survivor), respondent biological sex, all two- and three-way interactions, as well as the set of covariates including the individual’s baseline score on the outcome.

•Random intercepts will model interdependence within families

•Models predicting dichotomous outcomes (CSE and SSE yes/no, any indoor tanning) will be estimated using binary logistic multilevel models.

•Standard multilevel analysis will be conducted to predict SSE comprehensiveness sun protection behavior, perceived stress, physical activity, etc. using the same fixed and random effect parameters.

•Exploratory analyses of moderators of intervention efficacy will be conducted using this MLM approach by including each moderator (e.g., melanoma risk factors, month of the year enrolled) as an additional fixed effect factor in the model.”

12a-i) Imputation techniques to deal with attrition / missing values

Imputation techniques to deal with attrition / missing values: Not all participants will use the intervention/comparator as intended and attrition is typically high in ehealth trials. Specify how participants who did not use the application or dropped out from the trial were treated in the statistical analysis (a complete case analysis is strongly discouraged, and simple imputation techniques such as LOCF may also be problematic [4]).

	1	2	3	4	5	
subitem not at all important	○	○	○	○	○	essential

Does your paper address subitem 12a-i? *

Copy and paste relevant sections from the manuscript (include quotes in quotation marks "like this" to indicate direct quotes from your manuscript), or elaborate on this item by providing additional information not in the ms, or briefly explain why the item is not applicable/relevant for your study

“We will use an intent-to-treat approach to analyses of primary and secondary outcomes. Our approach to recruitment and retention minimizes missing data by contacting participants who do not complete sections of the survey, and by incentivizing participation. Initial analyses will examine characteristics of non-completers. As recommended by Lang & Little [76], multiple imputation (using 50 imputed samples) will be used to impute missing values.”

12b) Methods for additional analyses, such as subgroup analyses and adjusted analyses

Does your paper address CONSORT subitem 12b? *

Copy and paste relevant sections from the manuscript (include quotes in quotation marks "like this" to indicate direct quotes from your manuscript), or elaborate on this item by providing additional information not in the ms, or briefly explain why the item is not applicable/relevant for your study

“Mediation analyses will assess whether the effects of the intervention on outcomes are mediated by changes in family and normative factors (family discussion, family norms, family benefits) and individual attitudes (e.g., risk, benefits, barriers, self-efficacy, action planning). MLM will be used to estimate the key mediation paths using four component steps [77]. These MLMs will include dummy-coded variables to denote the combination of biological sex and participant role (e.g., mother, father), each of these dummy variables will also interact with treatment condition, and the overall intercept in this type of simple slopes model is suppressed. To assess mediation (see Figure 4) these predictors are included in a model that predicts the proposed mediator and the coefficients from the interaction between the dummy variables and condition estimate the “a” path for each role. To assess the “b” paths, the interactions between the dummy variables and the mediator are added to the model and the outcome (CSE, SSE, or sun protection) is predicted. The contribution of the mediated effect will be assessed directly using the approach recommended by Kenny and colleagues [78] for multilevel models. Specifically, the product of the two pieces of the mediating path (i.e., “a*b” from intervention group assignment to mediating variable and from mediating variable to outcome) will be calculated. Bootstrap procedures will be utilized to test for significance of the mediating pathway. “

X26) REB/IRB Approval and Ethical Considerations [recommended as subheading under "Methods"] (not a CONSORT item)

X26-i) Comment on ethics committee approval

	1	2	3	4	5	
subitem not at all important	○	○	○	●	○	essential

Clear selection

Does your paper address subitem X26-i?

Copy and paste relevant sections from the manuscript (include quotes in quotation marks "like this" to indicate direct quotes from your manuscript), or elaborate on this item by providing additional information not in the ms, or briefly explain why the item is not applicable/relevant for your study

“Study procedures were approved by the Rutgers University IRB (Pro2019000158).”

x26-ii) Outline informed consent procedures

Outline informed consent procedures e.g., if consent was obtained offline or online (how? Checkbox, etc.?), and what information was provided (see 4a-ii). See [6] for some items to be included in informed consent documents.

	1	2	3	4	5	
subitem not at all important	○	○	○	○	◉	essential

Clear selection

Does your paper address subitem X26-ii?

Copy and paste relevant sections from the manuscript (include quotes in quotation marks "like this" to indicate direct quotes from your manuscript), or elaborate on this item by providing additional information not in the ms, or briefly explain why the item is not applicable/relevant for your study

“Once the prospective participant’s eligibility is confirmed, informed consent is obtained using an electronic consent form hosted on DatStat. Participants are informed of the study requirements, potential risks and benefits of study participation, and a breakdown of study compensation. After informed consent and baseline surveys, participants are assigned a study identification number. Both the survivor and at least one FDR must consent and complete a baseline survey in order to be randomized.”

X26-iii) Safety and security procedures

Safety and security procedures, incl. privacy considerations, and any steps taken to reduce the likelihood or detection of harm (e.g., education and training, availability of a hotline)

	1	2	3	4	5	
subitem not at all important	○	○	○	○	◉	essential

Clear selection

Does your paper address subitem X26-iii?

Copy and paste relevant sections from the manuscript (include quotes in quotation marks "like this" to indicate direct quotes from your manuscript), or elaborate on this item by providing additional information not in the ms, or briefly explain why the item is not applicable/relevant for your study

“An adverse event for this study is defined as: a) a participant dying between baseline and follow-up (not a study-related adverse event); b) a participant reporting distress associated with study participation; and/or c) if the participant has breached confidentiality in the Facebook group (e.g., sharing a group member’s post that contains their name outside of the Facebook group). Participants reporting distress will be contacted by the site Principal Investigator (PI). During this telephone contact, the causes and degree of distress will be discussed. If the site PI feels there is a need for referral of the participant for psychological intervention, they will identify providers near the participant and provide the referral information. Participants breaching confidentiality will be contacted to let them know that they cannot share posts outside of the group. All adverse events will be documented by the study staff and reviewed on a monthly basis by the study PI and the Project Coordinator and reported to the Safety Officer. The IRB and Rutgers CINJ’s Protocol Monitoring Committee reviews all adverse events. The NIH and Rutgers IRB will be notified immediately in the event of serious adverse reactions or any unanticipated (not mentioned in the consent form) occurrence of physical or psychological harm or unexpected threat to privacy (e.g., lost records) or safety of subjects.”

RESULTS

13a) For each group, the numbers of participants who were randomly assigned, received intended treatment, and were analysed for the primary outcome

NPT: The number of care providers or centers performing the intervention in each group and the number of patients treated by each care provider in each center

Does your paper address CONSORT subitem 13a? *

Copy and paste relevant sections from the manuscript (include quotes in quotation marks "like this" to indicate direct quotes from your manuscript), or elaborate on this item by providing additional information not in the ms, or briefly explain why the item is not applicable/relevant for your study

N/A Trial is still underway; therefore results are not available.

13b) For each group, losses and exclusions after randomisation, together with reasons

Does your paper address CONSORT subitem 13b? (NOTE: Preferably, this is shown in a CONSORT flow diagram) *

Copy and paste relevant sections from the manuscript (include quotes in quotation marks "like this" to indicate direct quotes from your manuscript), or elaborate on this item by providing additional information not in the ms, or briefly explain why the item is not applicable/relevant for your study

N/A Trial is still underway; therefore results are not available.

13b-i) Attrition diagram

Strongly recommended: An attrition diagram (e.g., proportion of participants still logging in or using the intervention/comparator in each group plotted over time, similar to a survival curve) or other figures or tables demonstrating usage/dose/engagement.

	1	2	3	4	5	
subitem not at all important	○	○	●	○	○	essential

Clear selection

Does your paper address subitem 13b-i?

Copy and paste relevant sections from the manuscript or cite the figure number if applicable (include quotes in quotation marks "like this" to indicate direct quotes from your manuscript), or elaborate on this item by providing additional information not in the ms, or briefly explain why the item is not applicable/relevant for your study

N/A Trial is still underway; therefore results are not available.

14a) Dates defining the periods of recruitment and follow-up

Does your paper address CONSORT subitem 14a? *

Copy and paste relevant sections from the manuscript (include quotes in quotation marks "like this" to indicate direct quotes from your manuscript), or elaborate on this item by providing additional information not in the ms, or briefly explain why the item is not applicable/relevant for your study

N/A Trial is still underway; and recruitment is still ongoing, therefore exact dates are not available.

14a-i) Indicate if critical “secular events” fell into the study period

Indicate if critical “secular events” fell into the study period, e.g., significant changes in Internet resources available or “changes in computer hardware or Internet delivery resources”

	1	2	3	4	5	
subitem not at all important	○	◉	○	○	○	essential

Clear selection

Does your paper address subitem 14a-i?

Copy and paste relevant sections from the manuscript (include quotes in quotation marks "like this" to indicate direct quotes from your manuscript), or elaborate on this item by providing additional information not in the ms, or briefly explain why the item is not applicable/relevant for your study

N/A Trial is still underway; therefore results are not available.

14b) Why the trial ended or was stopped (early)

Does your paper address CONSORT subitem 14b? *

Copy and paste relevant sections from the manuscript (include quotes in quotation marks "like this" to indicate direct quotes from your manuscript), or elaborate on this item by providing additional information not in the ms, or briefly explain why the item is not applicable/relevant for your study

N/A Trial is still underway; therefore results are not available.

15) A table showing baseline demographic and clinical characteristics for each group

NPT: When applicable, a description of care providers (case volume, qualification, expertise, etc.) and centers (volume) in each group

Does your paper address CONSORT subitem 15? *

Copy and paste relevant sections from the manuscript (include quotes in quotation marks "like this" to indicate direct quotes from your manuscript), or elaborate on this item by providing additional information not in the ms, or briefly explain why the item is not applicable/relevant for your study

N/A Trial is still underway; therefore results are not available.

15-i) Report demographics associated with digital divide issues

In ehealth trials it is particularly important to report demographics associated with digital divide issues, such as age, education, gender, social-economic status, computer/Internet/ehealth literacy of the participants, if known.

	1	2	3	4	5	
subitem not at all important	○	○	○	○	○	essential

Does your paper address subitem 15-i? *

Copy and paste relevant sections from the manuscript (include quotes in quotation marks "like this" to indicate direct quotes from your manuscript), or elaborate on this item by providing additional information not in the ms, or briefly explain why the item is not applicable/relevant for your study

N/A Trial is still underway; therefore results are not available.

16) For each group, number of participants (denominator) included in each analysis and whether the analysis was by original assigned groups

16-i) Report multiple “denominators” and provide definitions

Report multiple “denominators” and provide definitions: Report N’s (and effect sizes) “across a range of study participation [and use] thresholds” [1], e.g., N exposed, N consented, N used more than x times, N used more than y weeks, N participants “used” the intervention/comparator at specific pre-defined time points of interest (in absolute and relative numbers per group). Always clearly define “use” of the intervention.

	1	2	3	4	5	
subitem not at all important	○	○	○	◉	○	essential

Clear selection

Does your paper address subitem 16-i? *

Copy and paste relevant sections from the manuscript (include quotes in quotation marks "like this" to indicate direct quotes from your manuscript), or elaborate on this item by providing additional information not in the ms, or briefly explain why the item is not applicable/relevant for your study

N/A Trial is still underway; therefore results are not available.

16-ii) Primary analysis should be intent-to-treat

Primary analysis should be intent-to-treat, secondary analyses could include comparing only “users”, with the appropriate caveats that this is no longer a randomized sample (see 18-i).

	1	2	3	4	5	
subitem not at all important	○	○	○	○	◉	essential

Clear selection

Does your paper address subitem 16-ii?

Copy and paste relevant sections from the manuscript (include quotes in quotation marks "like this" to indicate direct quotes from your manuscript), or elaborate on this item by providing additional information not in the ms, or briefly explain why the item is not applicable/relevant for your study

N/A Trial is still underway; therefore results are not available.

17a) For each primary and secondary outcome, results for each group, and the estimated effect size and its precision (such as 95% confidence interval)

Does your paper address CONSORT subitem 17a? *

Copy and paste relevant sections from the manuscript (include quotes in quotation marks "like this" to indicate direct quotes from your manuscript), or elaborate on this item by providing additional information not in the ms, or briefly explain why the item is not applicable/relevant for your study

N/A Trial is still underway; therefore results are not available.

17a-i) Presentation of process outcomes such as metrics of use and intensity of use

In addition to primary/secondary (clinical) outcomes, the presentation of process outcomes such as metrics of use and intensity of use (dose, exposure) and their operational definitions is critical. This does not only refer to metrics of attrition (13-b) (often a binary variable), but also to more continuous exposure metrics such as "average session length". These must be accompanied by a technical description how a metric like a "session" is defined (e.g., timeout after idle time) [1] (report under item 6a).

	1	2	3	4	5	
subitem not at all important	○	○	○	◉	○	essential

Clear selection

Does your paper address subitem 17a-i?

Copy and paste relevant sections from the manuscript (include quotes in quotation marks "like this" to indicate direct quotes from your manuscript), or elaborate on this item by providing additional information not in the ms, or briefly explain why the item is not applicable/relevant for your study

N/A Trial is still underway; therefore results are not available.

17b) For binary outcomes, presentation of both absolute and relative effect sizes is recommended

Does your paper address CONSORT subitem 17b? *

Copy and paste relevant sections from the manuscript (include quotes in quotation marks "like this" to indicate direct quotes from your manuscript), or elaborate on this item by providing additional information not in the ms, or briefly explain why the item is not applicable/relevant for your study

N/A Trial is still underway; therefore results are not available.

18) Results of any other analyses performed, including subgroup analyses and adjusted analyses, distinguishing pre-specified from exploratory

Does your paper address CONSORT subitem 18? *

Copy and paste relevant sections from the manuscript (include quotes in quotation marks "like this" to indicate direct quotes from your manuscript), or elaborate on this item by providing additional information not in the ms, or briefly explain why the item is not applicable/relevant for your study

N/A Trial is still underway; therefore results are not available.

18-i) Subgroup analysis of comparing only users

A subgroup analysis of comparing only users is not uncommon in ehealth trials, but if done, it must be stressed that this is a self-selected sample and no longer an unbiased sample from a randomized trial (see 16-iii).

	1	2	3	4	5	
subitem not at all important	○	◉	○	○	○	essential

Clear selection

Does your paper address subitem 18-i?

Copy and paste relevant sections from the manuscript (include quotes in quotation marks "like this" to indicate direct quotes from your manuscript), or elaborate on this item by providing additional information not in the ms, or briefly explain why the item is not applicable/relevant for your study

N/A Trial is still underway; therefore results are not available.

19) All important harms or unintended effects in each group

(for specific guidance see CONSORT for harms)

Does your paper address CONSORT subitem 19? *

Copy and paste relevant sections from the manuscript (include quotes in quotation marks "like this" to indicate direct quotes from your manuscript), or elaborate on this item by providing additional information not in the ms, or briefly explain why the item is not applicable/relevant for your study

N/A Trial is still underway; therefore results are not available. As of now there have been no harms reported

19-i) Include privacy breaches, technical problems

Include privacy breaches, technical problems. This does not only include physical “harm” to participants, but also incidents such as perceived or real privacy breaches [1], technical problems, and other unexpected/unintended incidents. “Unintended effects” also includes unintended positive effects [2].

	1	2	3	4	5	
subitem not at all important	○	○	○	●	○	essential

Clear selection

Does your paper address subitem 19-i?

Copy and paste relevant sections from the manuscript (include quotes in quotation marks "like this" to indicate direct quotes from your manuscript), or elaborate on this item by providing additional information not in the ms, or briefly explain why the item is not applicable/relevant for your study

N/A Trial is still underway; therefore results are not available. As of now there have been no breaches reported

19-ii) Include qualitative feedback from participants or observations from staff/researchers

Include qualitative feedback from participants or observations from staff/researchers, if available, on strengths and shortcomings of the application, especially if they point to unintended/unexpected effects or uses. This includes (if available) reasons for why people did or did not use the application as intended by the developers.

	1	2	3	4	5	
subitem not at all important	○	●	○	○	○	essential

Clear selection

Does your paper address subitem 19-ii?

Copy and paste relevant sections from the manuscript (include quotes in quotation marks "like this" to indicate direct quotes from your manuscript), or elaborate on this item by providing additional information not in the ms, or briefly explain why the item is not applicable/relevant for your study

N/A Trial is still underway; therefore results are not available.

DISCUSSION

22) Interpretation consistent with results, balancing benefits and harms, and considering other relevant evidence

NPT: In addition, take into account the choice of the comparator, lack of or partial blinding, and unequal expertise of care providers or centers in each group

22-i) Restate study questions and summarize the answers suggested by the data, starting with primary outcomes and process outcomes (use)

Restate study questions and summarize the answers suggested by the data, starting with primary outcomes and process outcomes (use).

	1	2	3	4	5	
subitem not at all important	○	○	○	○	◉	essential

Clear selection

Does your paper address subitem 22-i? *

Copy and paste relevant sections from the manuscript (include quotes in quotation marks "like this" to indicate direct quotes from your manuscript), or elaborate on this item by providing additional information not in the ms, or briefly explain why the item is not applicable/relevant for your study

N/A Trial is still underway; therefore results and full discussion are not available.

22-ii) Highlight unanswered new questions, suggest future research

Highlight unanswered new questions, suggest future research.

	1	2	3	4	5	
subitem not at all important	○	○	○	○	◉	essential

Clear selection

Does your paper address subitem 22-ii?

Copy and paste relevant sections from the manuscript (include quotes in quotation marks "like this" to indicate direct quotes from your manuscript), or elaborate on this item by providing additional information not in the ms, or briefly explain why the item is not applicable/relevant for your study

N/A Trial is still underway; therefore results and full discussion are not available. However, we provide potential avenues for future research, as seen below:
“The impact of this trial lies in its implementation and dissemination potential in a variety of contexts given the use of a familiar, popular, freely-available social media platform. We will produce a content library of posts and a user guide that can guide implementation by others. If efficacious, the intervention could be disseminated and delivered via dermatologist practices, public health organizations such as the American Cancer Society, non-profit organizations focused on melanoma, or in partnership with existing melanoma and skin cancer Facebook groups. The ability to interact with a skin cancer expert and other families affected by melanoma synchronously and asynchronously on Facebook may not only be convenient, but it may also be interesting to participants and lead to a more impactful intervention. Knowledge gained will provide important information about potentially cost-effective ways to reach survivors and their families. If efficacious, future research should evaluate potential implementation strategies to reach young survivors and their family members with social media interventions.

20) Trial limitations, addressing sources of potential bias, imprecision, and, if relevant, multiplicity of analyses

20-i) Typical limitations in ehealth trials

Typical limitations in ehealth trials: Participants in ehealth trials are rarely blinded. Ehealth trials often look at a multiplicity of outcomes, increasing risk for a Type I error. Discuss biases due to non-use of the intervention/usability issues, biases through informed consent procedures, unexpected events.

	1	2	3	4	5	
subitem not at all important	○	○	○	◉	○	essential

Clear selection

Does your paper address subitem 20-i? *

Copy and paste relevant sections from the manuscript (include quotes in quotation marks "like this" to indicate direct quotes from your manuscript), or elaborate on this item by providing additional information not in the ms, or briefly explain why the item is not applicable/relevant for your study

“First, Facebook posts tend to be brief, which may not be conducive to explaining complex concepts. Although videos can be used to explain complex concepts, many Facebook users may not view lengthy videos. Further, some group members may not engage in the group (e.g., “lurk”) or view all posts. Studies of Facebook interventions have shown that total group engagement tends to decline over time [63]. We leverage a number of strategies to enhance engagement including using polls, conversation starters, and Questions and Answers with experts in the Facebook posts. Future work may need to include only regular users of Facebook. Second, recruiting a large enough number of participants to populate multi-family groups of 50-60 family members within a relatively short period of time can be challenging. Timely enrollment is important when enrolling a cohort of 100 participants to randomize into two groups. Finally, although self-report sun protection measures show moderate correspondence with behavioral observations and diaries [86, 87] and have moderate to high reproducibility and internal consistency [88-90] .”

21) Generalisability (external validity, applicability) of the trial findings

NPT: External validity of the trial findings according to the intervention, comparators, patients, and care providers or centers involved in the trial

21-i) Generalizability to other populations

Generalizability to other populations: In particular, discuss generalizability to a general Internet population, outside of a RCT setting, and general patient population, including applicability of the study results for other organizations

	1	2	3	4	5	
subitem not at all important	○	○	○	◉	○	essential

Clear selection

Does your paper address subitem 21-i?

Copy and paste relevant sections from the manuscript (include quotes in quotation marks "like this" to indicate direct quotes from your manuscript), or elaborate on this item by providing additional information not in the ms, or briefly explain why the item is not applicable/relevant for your study

N/A Trial is still underway; therefore results and full discussion are not available.

21-ii) Discuss if there were elements in the RCT that would be different in a routine application setting

Discuss if there were elements in the RCT that would be different in a routine application setting (e.g., prompts/reminders, more human involvement, training sessions or other co-interventions) and what impact the omission of these elements could have on use, adoption, or outcomes if the intervention is applied outside of a RCT setting.

	1	2	3	4	5	
subitem not at all important	○	◉	○	○	○	essential

Clear selection

Does your paper address subitem 21-ii?

Copy and paste relevant sections from the manuscript (include quotes in quotation marks "like this" to indicate direct quotes from your manuscript), or elaborate on this item by providing additional information not in the ms, or briefly explain why the item is not applicable/relevant for your study

N/A Trial is still underway; therefore results and full discussion are not available.

OTHER INFORMATION

23) Registration number and name of trial registry

Does your paper address CONSORT subitem 23? *

Copy and paste relevant sections from the manuscript (include quotes in quotation marks "like this" to indicate direct quotes from your manuscript), or elaborate on this item by providing additional information not in the ms, or briefly explain why the item is not applicable/relevant for your study

“Trial Registration: ClinicalTrials.gov NCT03677739”

24) Where the full trial protocol can be accessed, if available

Does your paper address CONSORT subitem 24? *

Cite a Multimedia Appendix, other reference, or copy and paste relevant sections from the manuscript (include quotes in quotation marks "like this" to indicate direct quotes from your manuscript), or elaborate on this item by providing additional information not in the ms, or briefly explain why the item is not applicable/relevant for your study

“Data Sharing: The data sets generated during and/or analyzed during the current study are available from the corresponding author on reasonable request.”
This trial is still ongoing but is registered at clinicaltrials.gov, the full protocol document will be available there.

25) Sources of funding and other support (such as supply of drugs), role of funders

Does your paper address CONSORT subitem 25? *

Copy and paste relevant sections from the manuscript (include quotes in quotation marks "like this" to indicate direct quotes from your manuscript), or elaborate on this item by providing additional information not in the ms, or briefly explain why the item is not applicable/relevant for your study

“Funding and Support: This project was supported by the National Cancer Institute under R01 CA221854, awarded to Sharon Manne. Services, results and/or products in support of the research project were generated by Rutgers Cancer Institute of New Jersey Population Science Research Support Shared Resource.”

X27) Conflicts of Interest (not a CONSORT item)

X27-i) State the relation of the study team towards the system being evaluated

In addition to the usual declaration of interests (financial or otherwise), also state the relation of the study team towards the system being evaluated, i.e., state if the authors/evaluators are distinct from or identical with the developers/sponsors of the intervention.

	1	2	3	4	5	
subitem not at all important	○	○	○	◉	○	essential

Clear selection

Does your paper address subitem X27-i?

Copy and paste relevant sections from the manuscript (include quotes in quotation marks "like this" to indicate direct quotes from your manuscript), or elaborate on this item by providing additional information not in the ms, or briefly explain why the item is not applicable/relevant for your study

"Conflicts of Interest Disclosures: No potential competing interest was reported by the authors."

About the CONSORT EHEALTH checklist

As a result of using this checklist, did you make changes in your manuscript? *

- ○ yes, major changes
- ○ yes, minor changes
- ◉ no

What were the most important changes you made as a result of using this checklist?

N/A

How much time did you spend on going through the checklist INCLUDING making changes in your manuscript *

My team, myself included spent overall 10 hours on this.

As a result of using this checklist, do you think your manuscript has improved? *

- ○ yes
- ◉ no
- ○ Other:

Would you like to become involved in the CONSORT EHEALTH group?

This would involve for example becoming involved in participating in a workshop and writing an "Explanation and Elaboration" document

- ○ yes
- ◉ no
- ○ Other:

Clear selection

Any other comments or questions on CONSORT EHEALTH

It is totally overkill. Many sections do not pertain to the manuscript that we are publishing at this time.

STOP - Save this form as PDF before you click submit

To generate a record that you filled in this form, we recommend to generate a PDF of this page (on a Mac, simply select "print" and then select "print as PDF") before you submit it.

When you submit your (revised) paper to JMIR, please upload the PDF as supplementary file.

Don't worry if some text in the textboxes is cut off, as we still have the complete information in our database. Thank you!

Final step: Click submit !

Click submit so we have your answers in our database!

Submit Clear form

[illegible]

This content is neither created nor endorsed by Google. Report Abuse - Terms of Service - Privacy Policy

Google Forms
